# Supplementary material for: Pressure-induced symmetry changes in body-centred cubic zeolites
Source: R Soc Open Sci. 2019 Jul 24;6(7):182158. doi: 10.1098/rsos.182158 (PMC6689579; doi:10.1098/rsos.182158)
Supplement: Figures showing the raw and refined data [file rsos182158supp1.docx]

Pressure-induced symmetry changes in body-centred cubic zeolites – Supplementary Information

Antony Nearchou, Mero-Lee U. Cornelius, Zöe L. Jones, I. E. Collings, Stephen A. Wells, Paul R. Raithby and Asel Sartbaeva

**
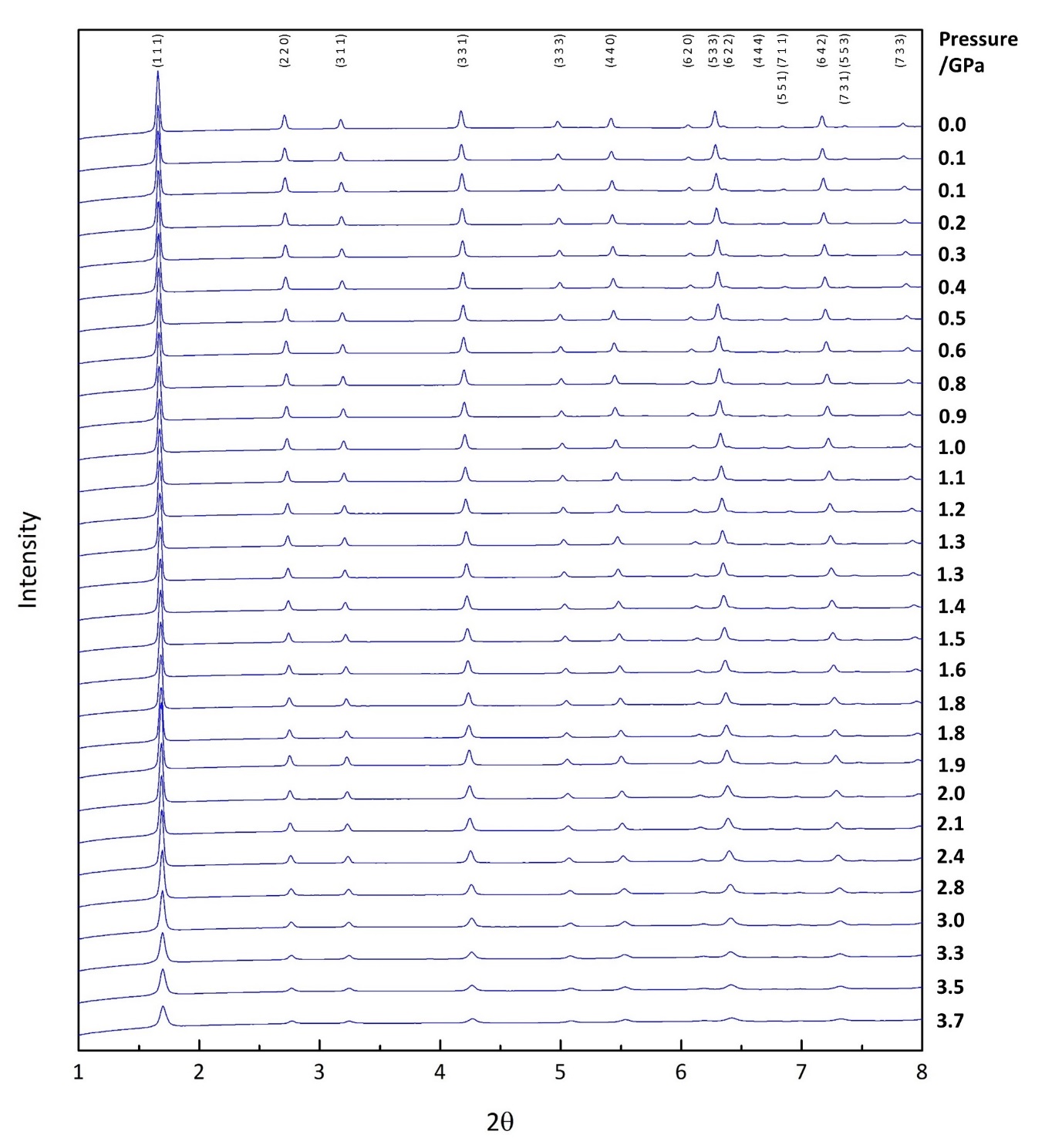
**

**Figure S1.** Powder X-ray diffraction patterns (2θ = 1-8°) of empty zeolite Na-X (FAU) as a function of pressure throughout the compression cycle.

**
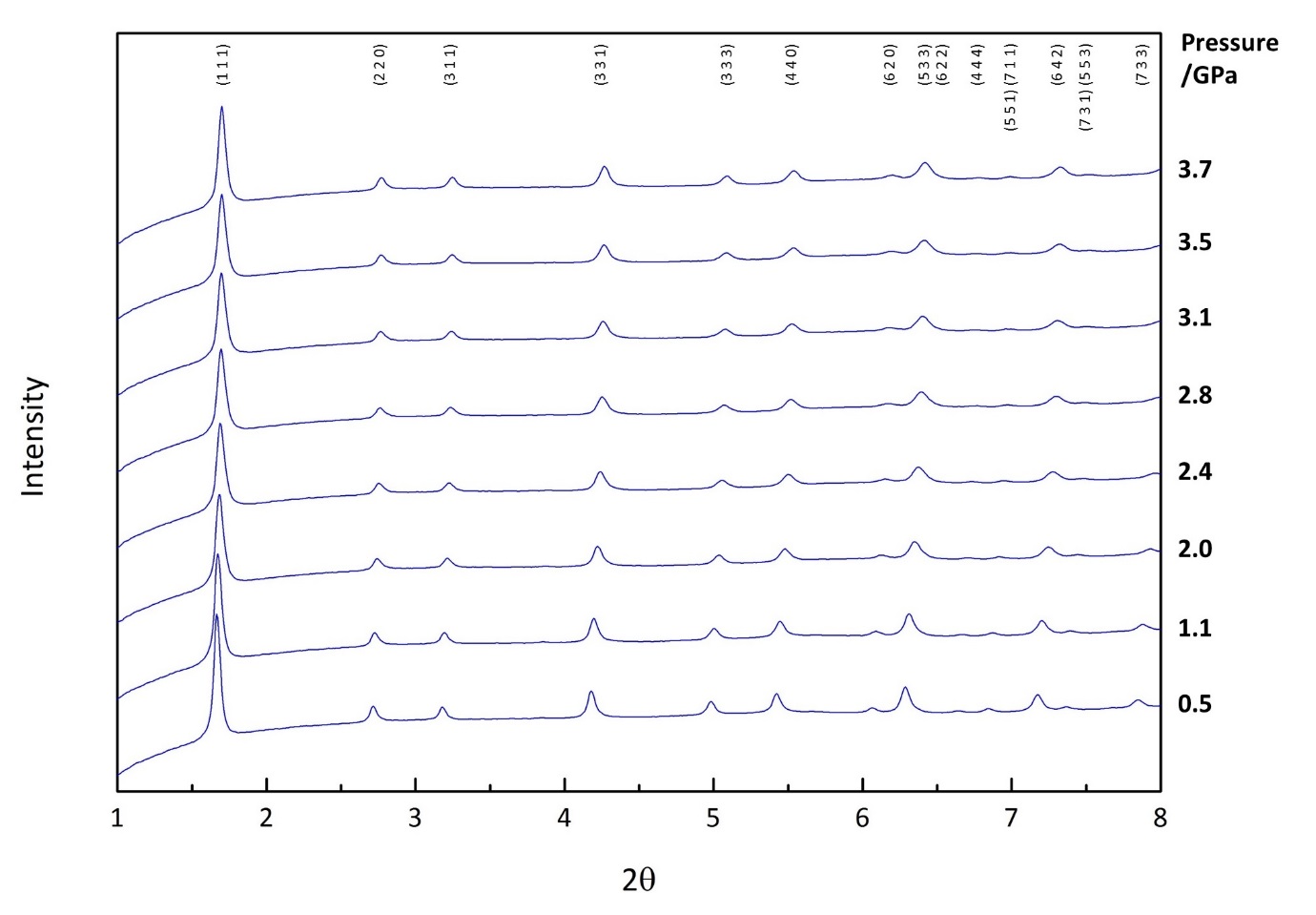
**

**Figure S2.** Powder X-ray diffraction patterns (2θ = 1-8°) of empty zeolite Na-X (FAU) as a function of pressure throughout the decompression cycle.


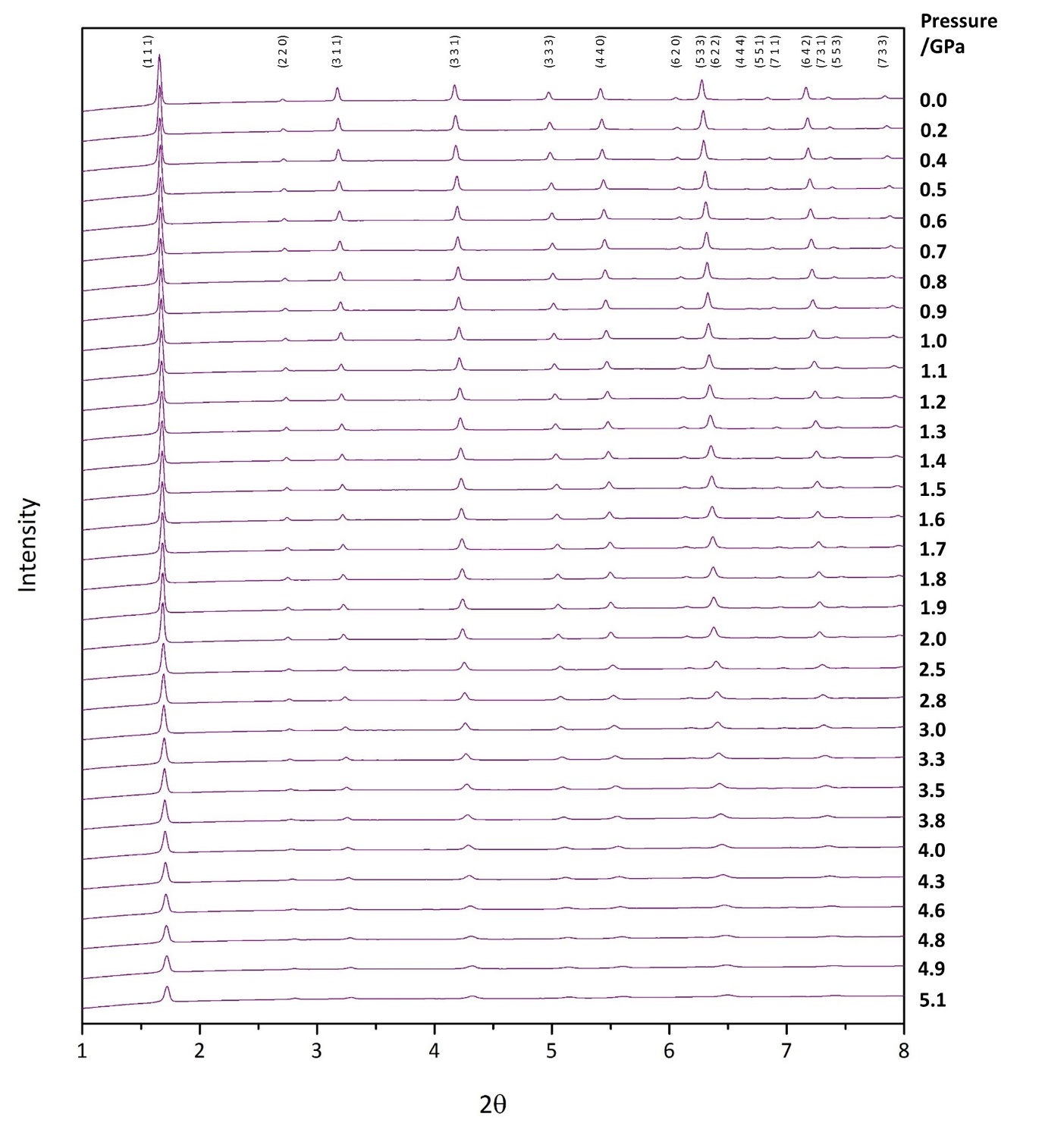


**Figure S3.** Powder X-ray diffraction patterns (2θ = 1-8°) of filled zeolite Na-X (FAU) as a function of pressure throughout the compression cycle.


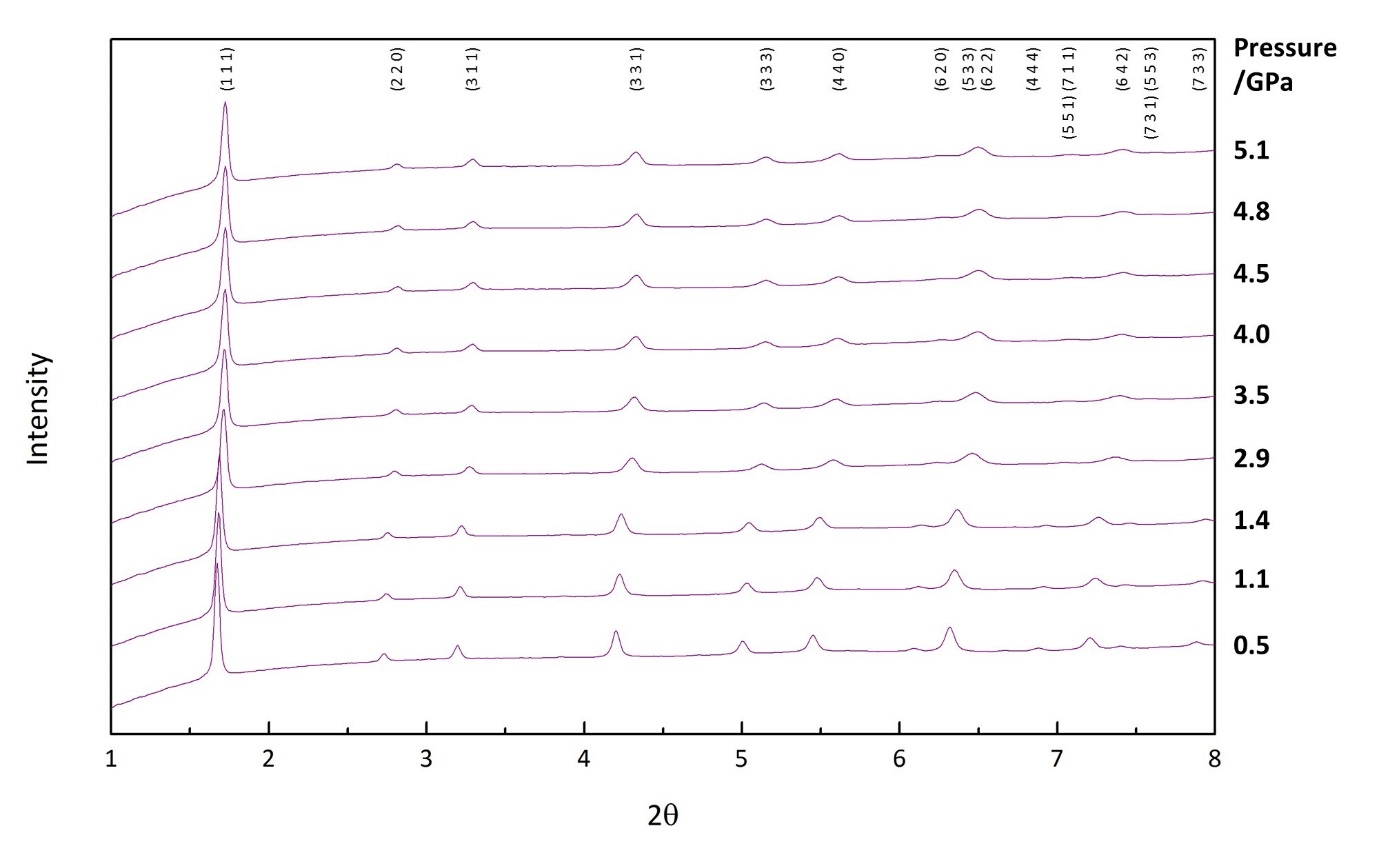


**Figure S4.** Powder X-ray diffraction patterns (2θ = 1-8°) of filled zeolite Na-X (FAU) as a function of pressure throughout the decompression cycle.


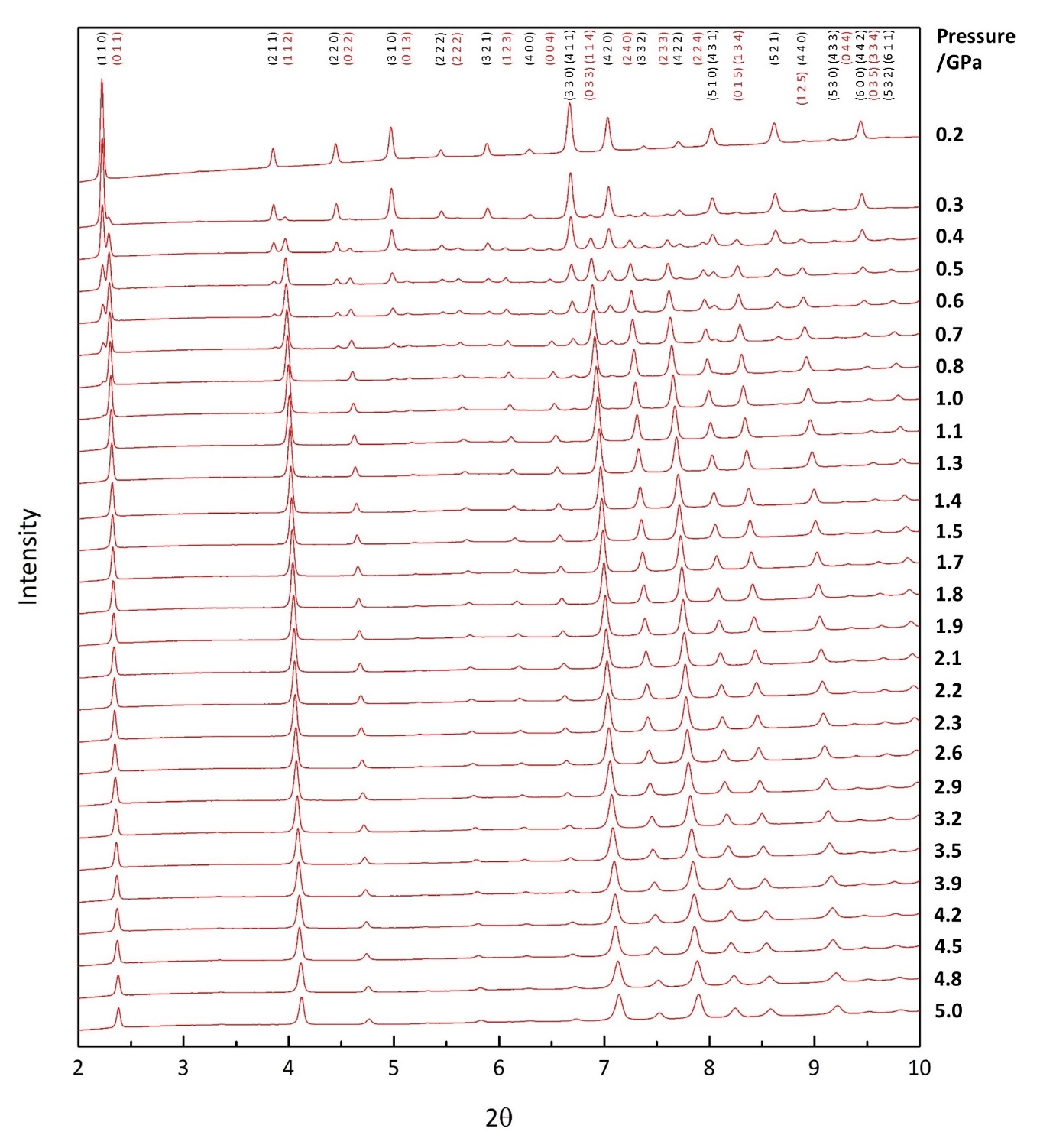


**Figure S5.** Powder X-ray diffraction patterns (2θ = 2-10°) of empty zeolite RHO (RHO) as a function of pressure throughout the compression cycle. Miller indices corresponding to the C-form are shown in black, and the A-form in red.

**
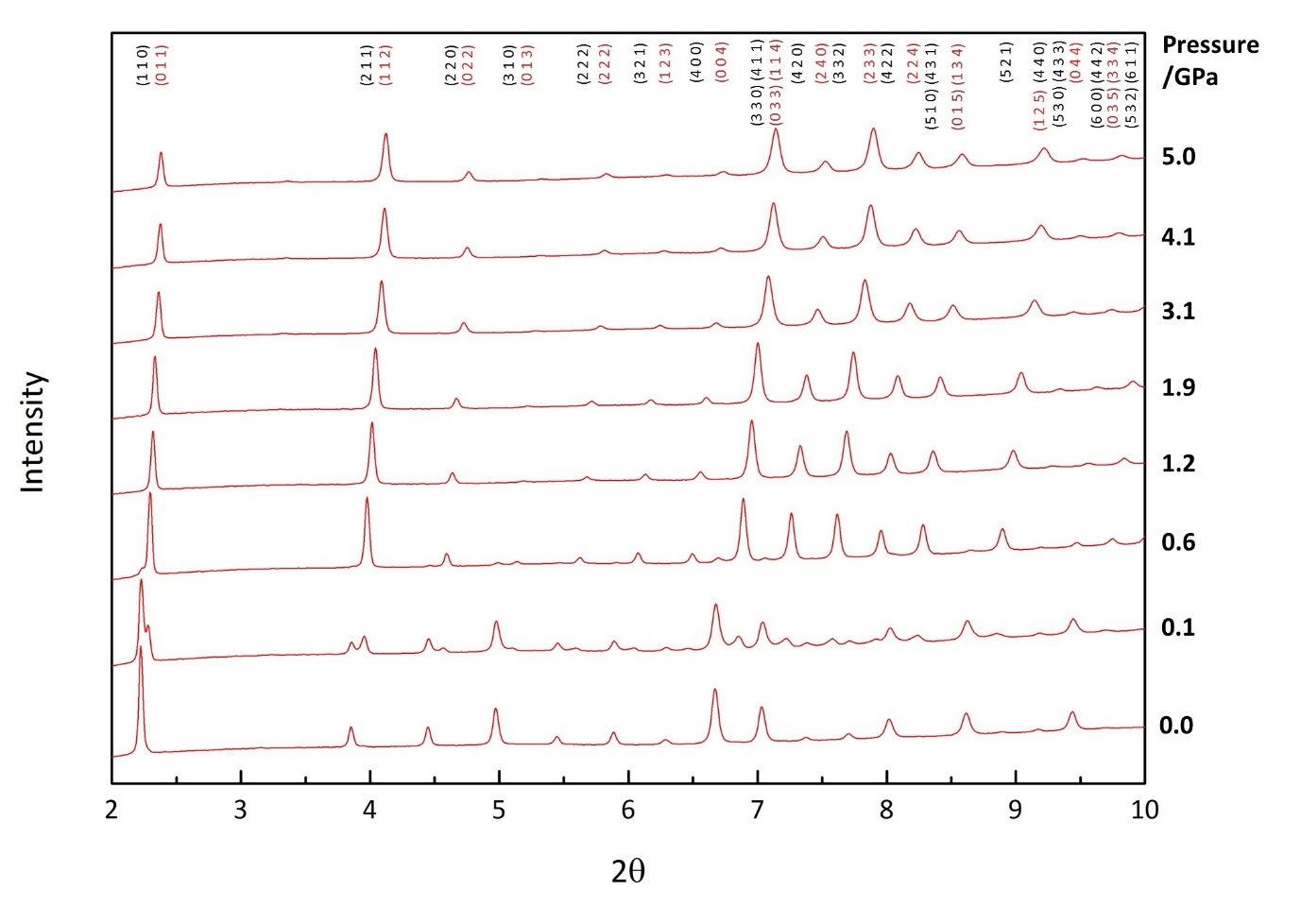
**

**Figure S6.** Powder X-ray diffraction patterns (2θ = 2-10°) of empty zeolite RHO (RHO) as a function of pressure throughout the decompression cycle. Miller indices corresponding to the C-form are shown in black, and the A-form in red.


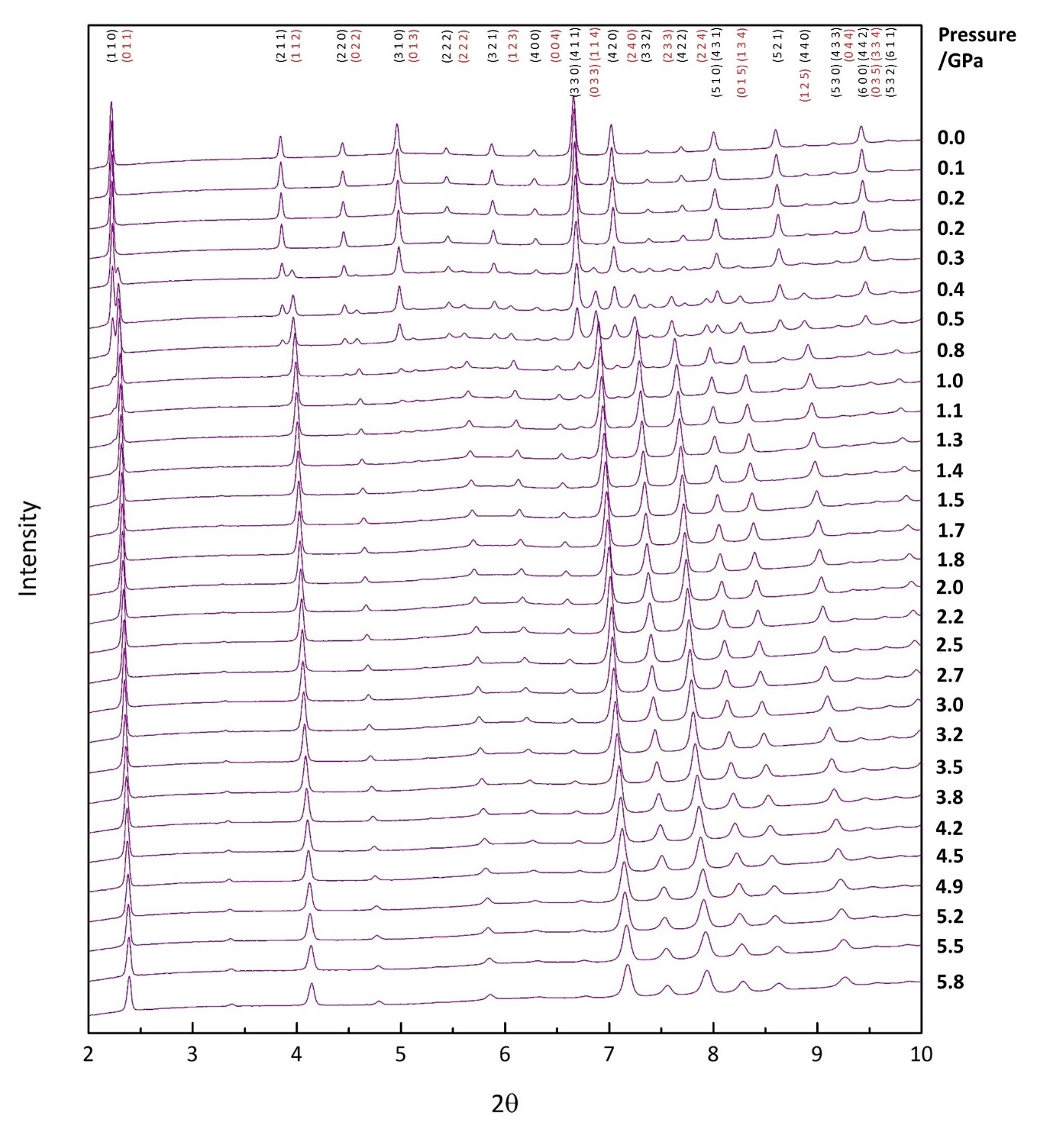


**Figure S7.** Powder X-ray diffraction patterns (2θ = 2-10°) of filled zeolite RHO (RHO) as a function of pressure throughout the compression cycle. Miller indices corresponding to the C-form are shown in black, and the A-form in red.

**
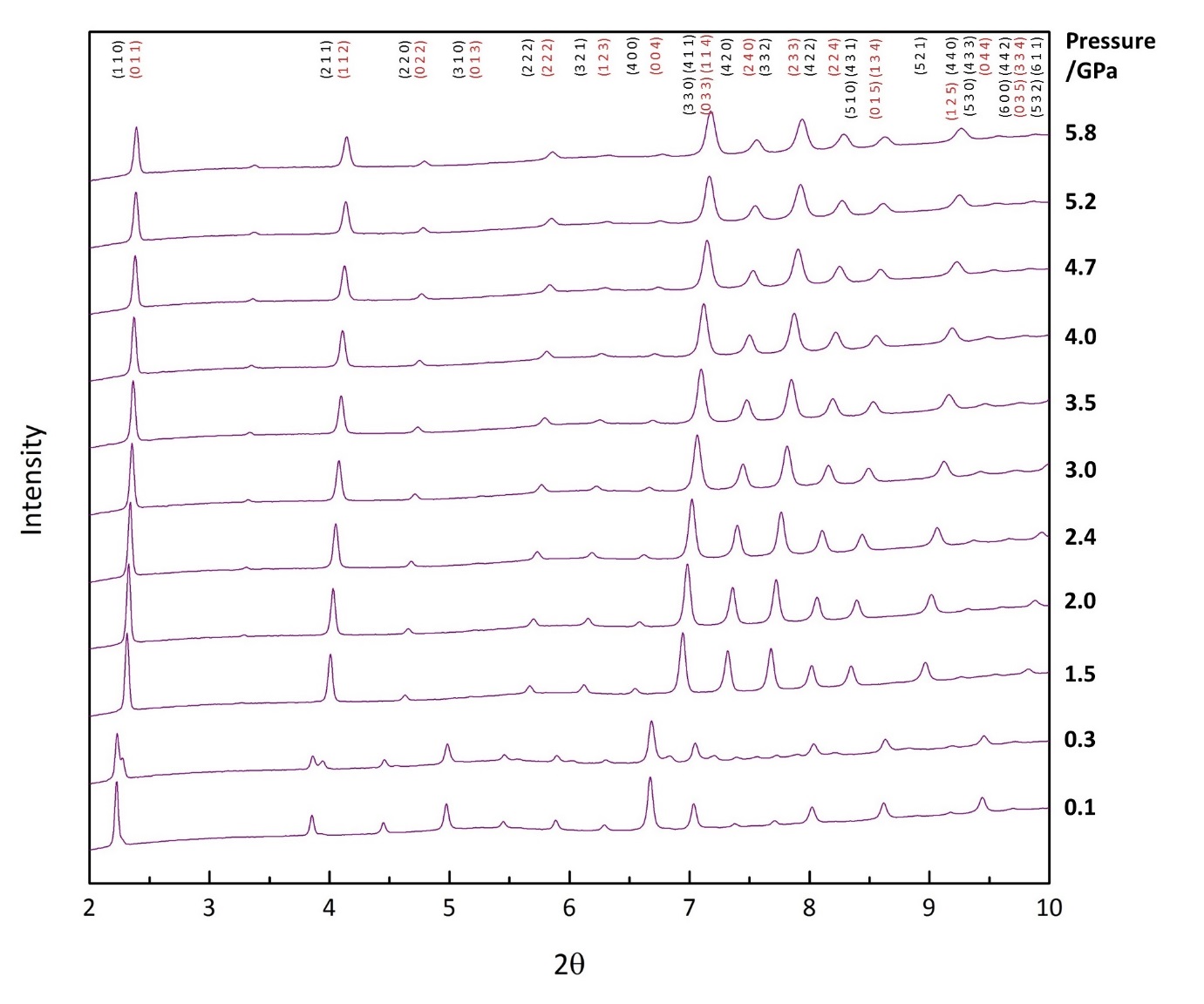
**

**Figure S8.** Powder X-ray diffraction patterns (2θ = 2-10°) of filled zeolite RHO (RHO) as a function of pressure throughout the decompression cycle. Miller indices corresponding to the C-form are shown in black, and the A-form in red.

**
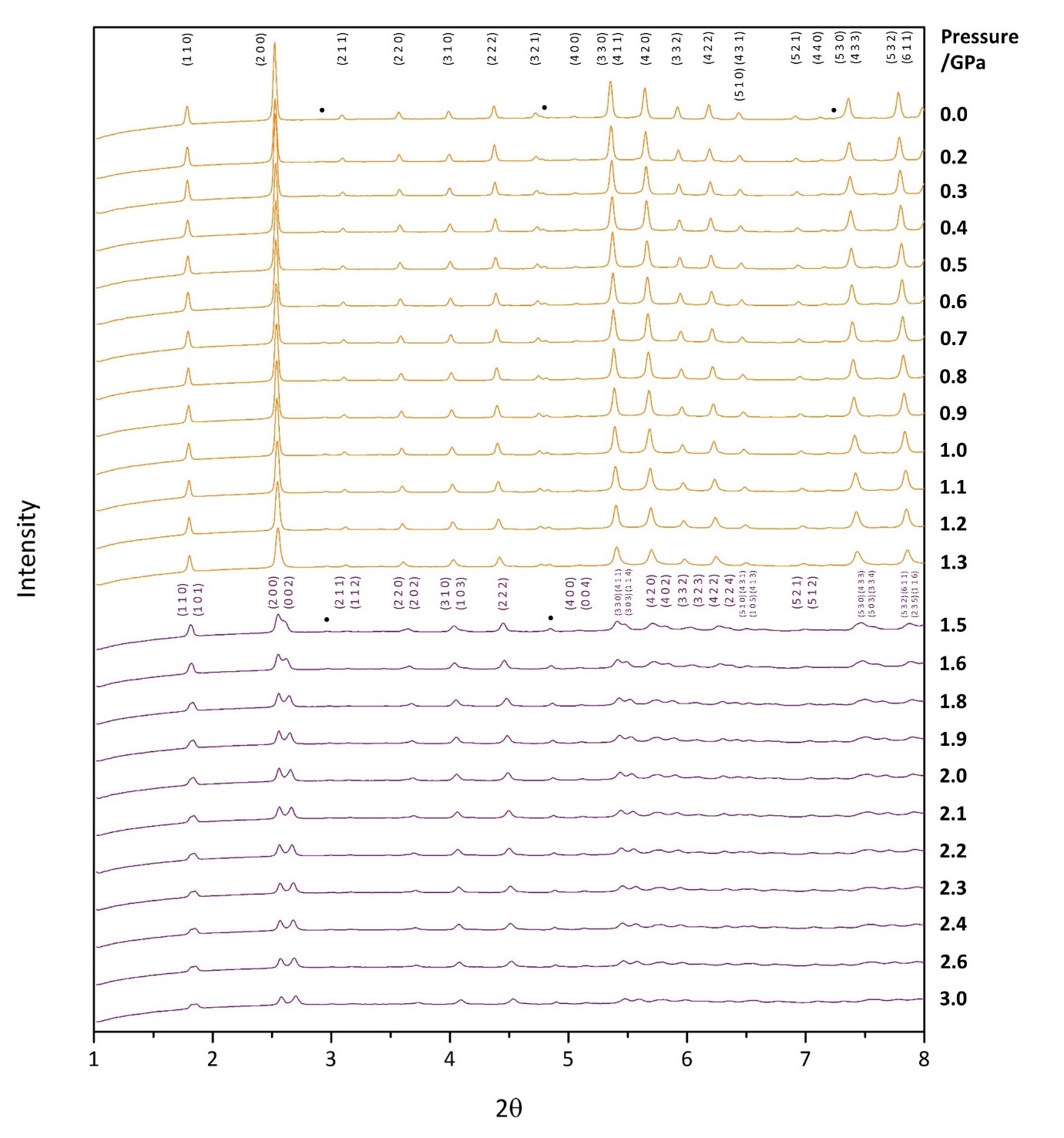
**

**Figure S9.** Powder X-ray diffraction patterns (2θ = 1-8°) of empty zeolite ZK-5 (KFI) as a function of pressure throughout the compression cycle. Patterns identified as the cubic form are shown in orange, and the tetragonal form in purple. Miller indices corresponding to the cubic form are shown in black, and the tetragonal form in purple. The dot refers to Bragg peaks from the zeolite W (MER) impurity phase.


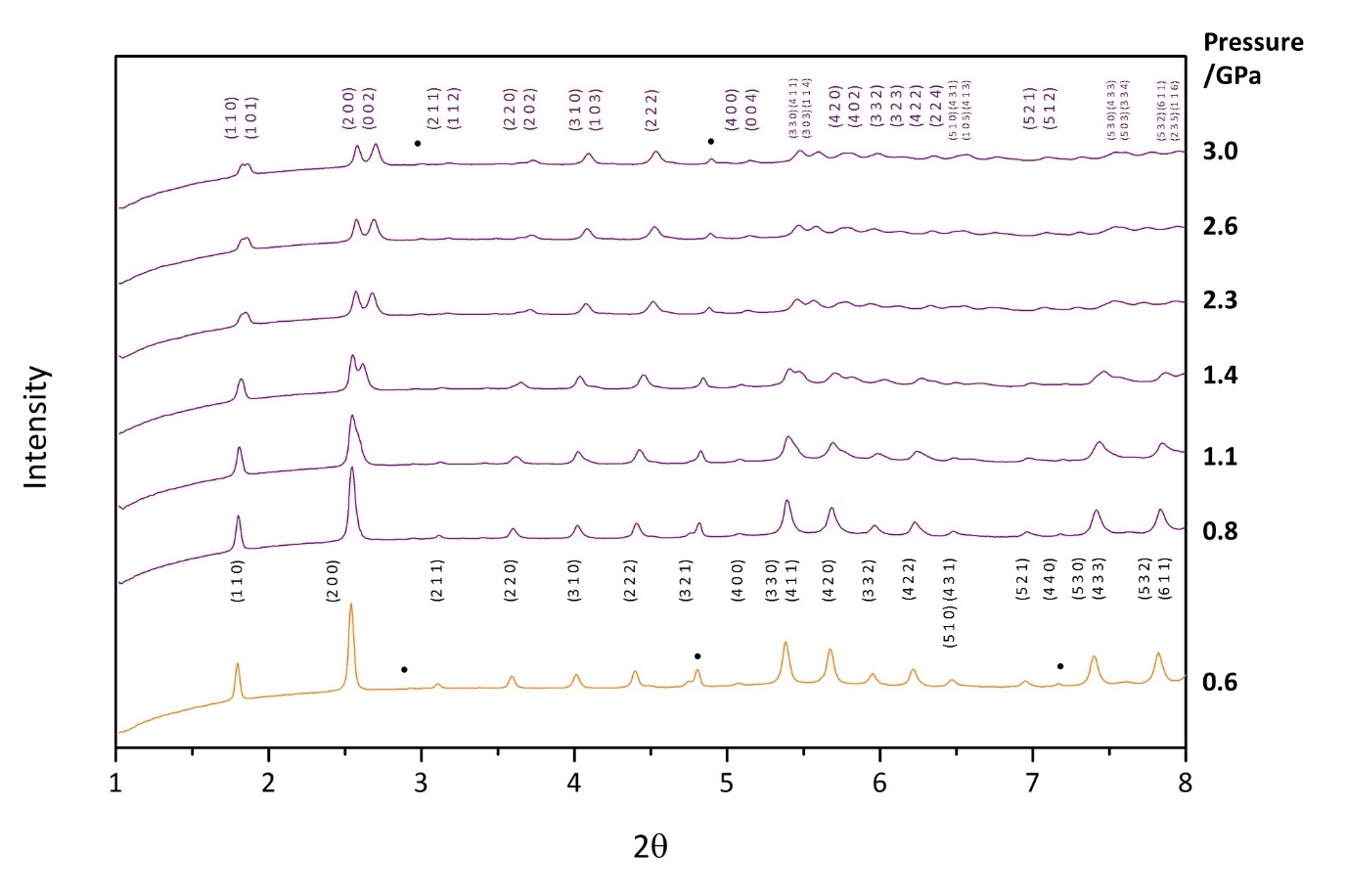


**Figure S10.** Powder X-ray diffraction patterns (2θ = 1-8°) of empty zeolite ZK-5 (KFI) as a function of pressure throughout the decompression cycle. Patterns identified as the cubic form are shown in orange, and the tetragonal form in purple. Miller indices corresponding to the cubic form are shown in black, and the tetragonal form in purple. The dot refers to Bragg peaks from the zeolite W (MER) impurity phase.

**
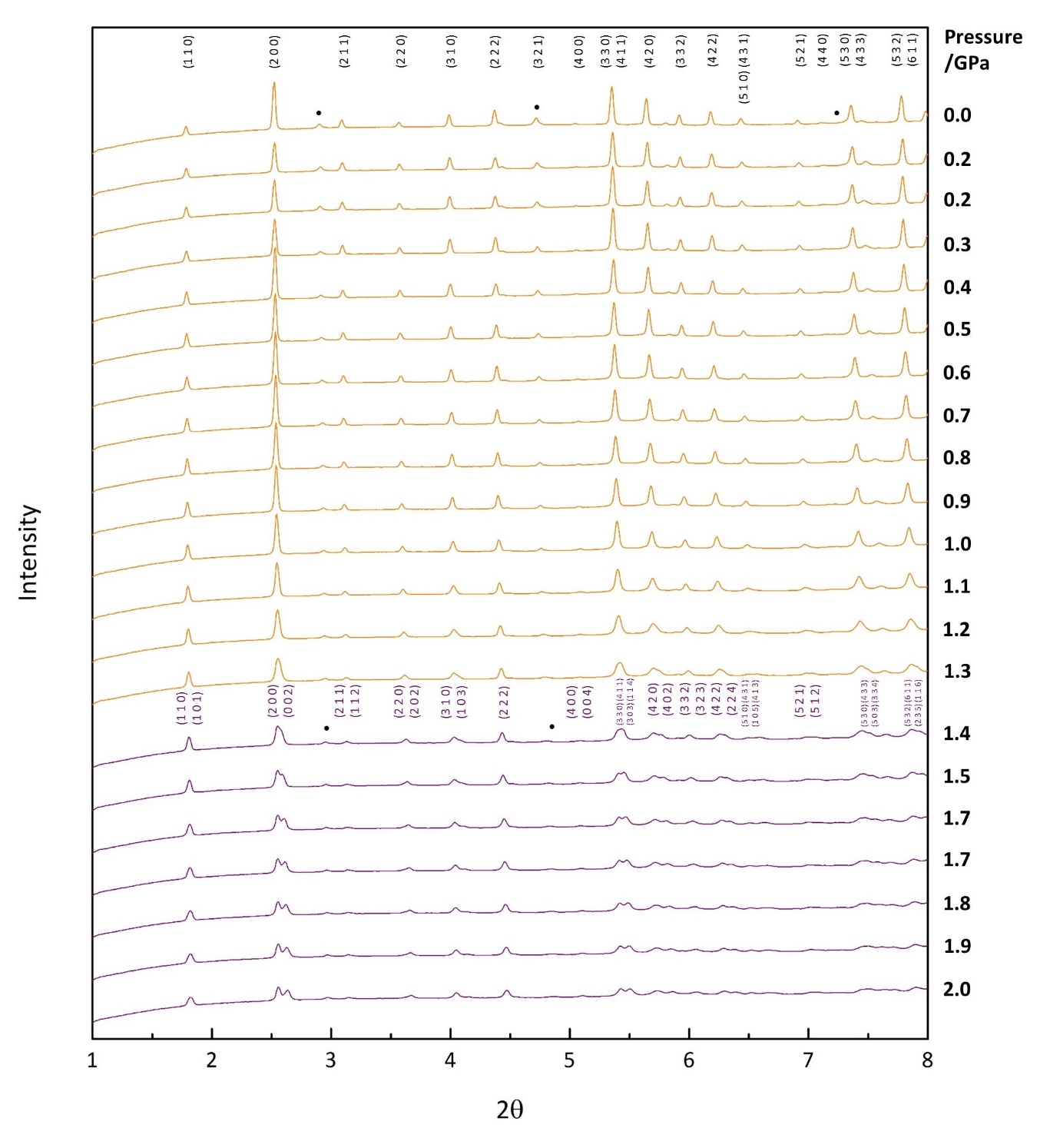
**

**Figure S11.** Powder X-ray diffraction patterns (2θ = 1-8°) of filled zeolite ZK-5 (KFI) as a function of pressure throughout the compression cycle. Patterns identified as the cubic form are shown in orange, and the tetragonal form in purple. Miller indices corresponding to the cubic form are shown in black, and the tetragonal form in purple. The dot refers to Bragg peaks from the zeolite W (MER) impurity phase.

**
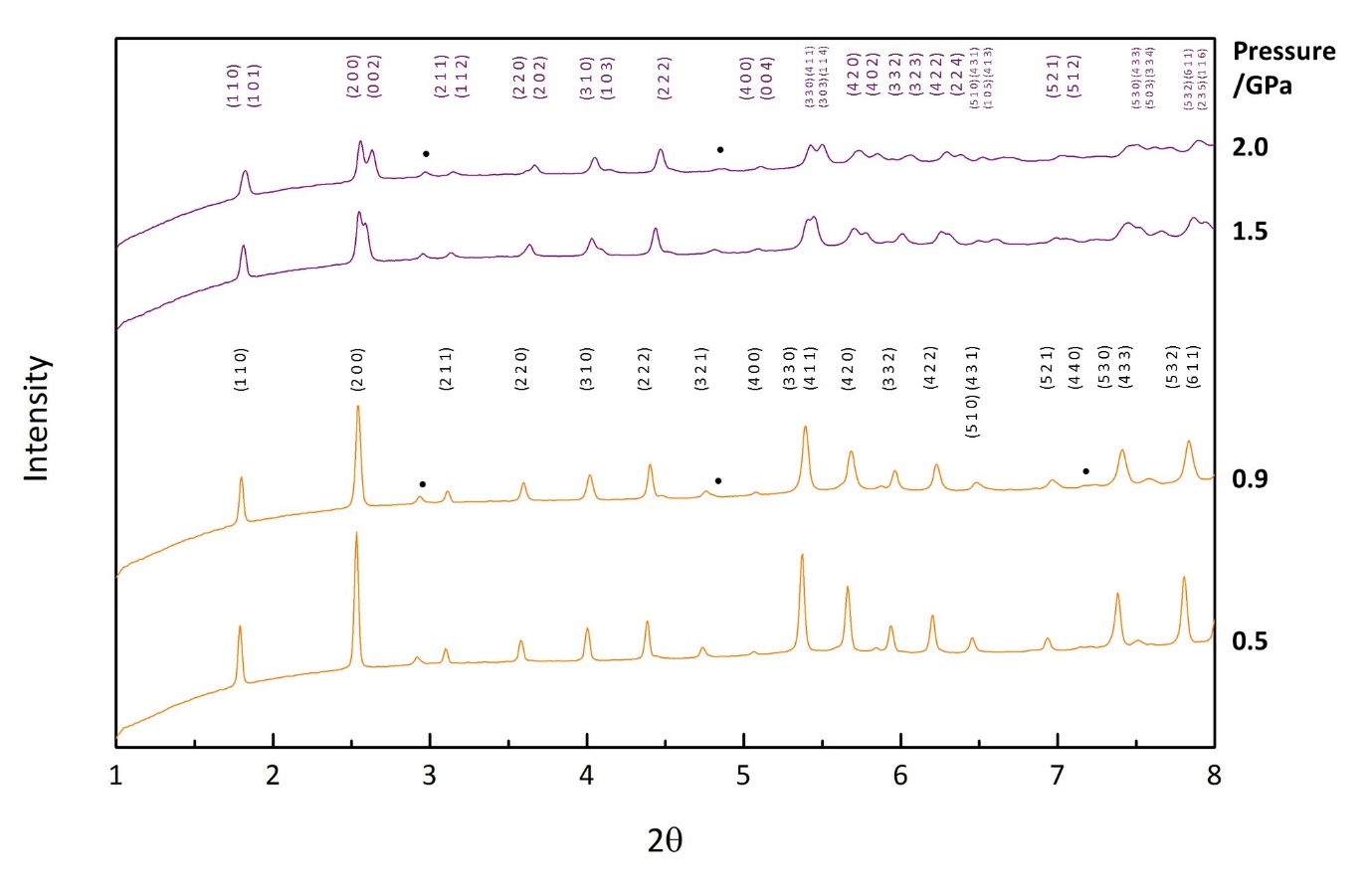
**

**Figure S12.** Powder X-ray diffraction patterns (2θ = 1-8°) of filled zeolite ZK-5 (KFI) as a function of pressure throughout the decompression cycle. Patterns identified as the cubic form are shown in orange, and the tetragonal form in purple. Miller indices corresponding to the cubic form are shown in black, and the tetragonal form in purple. The dot refers to Bragg peaks from the zeolite W (MER) impurity phase.

**
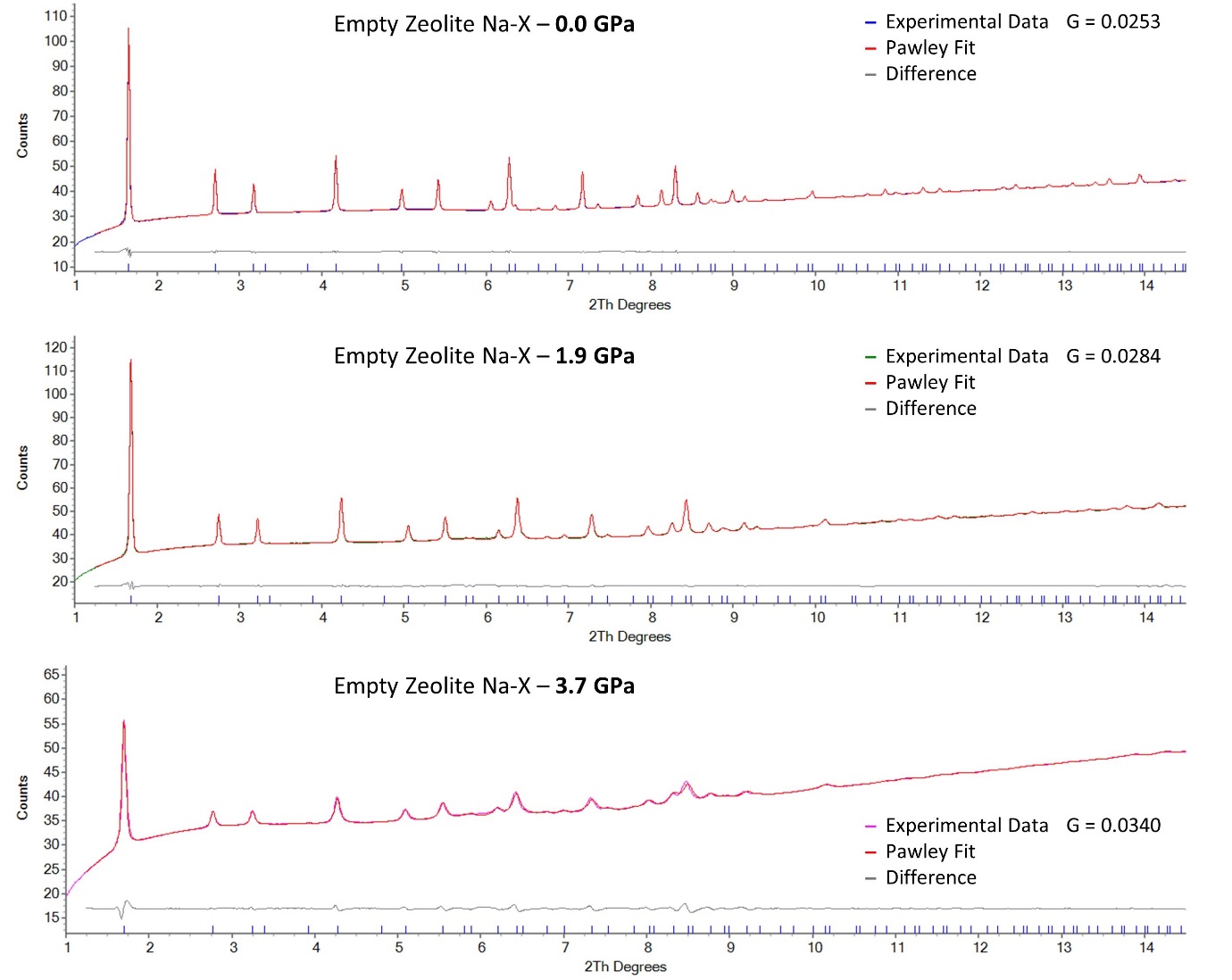
**

**Figure S13.** Final refined Pawley fits for empty zeolite Na-X (FAU) at pressures of 0.0, 1.9 and 3.7 GPa. The patterns in blue, green and pink correspond to the experimental data, red to the Pawley fit and grey to the difference. G refers to the ‘goodness of fit’.

**
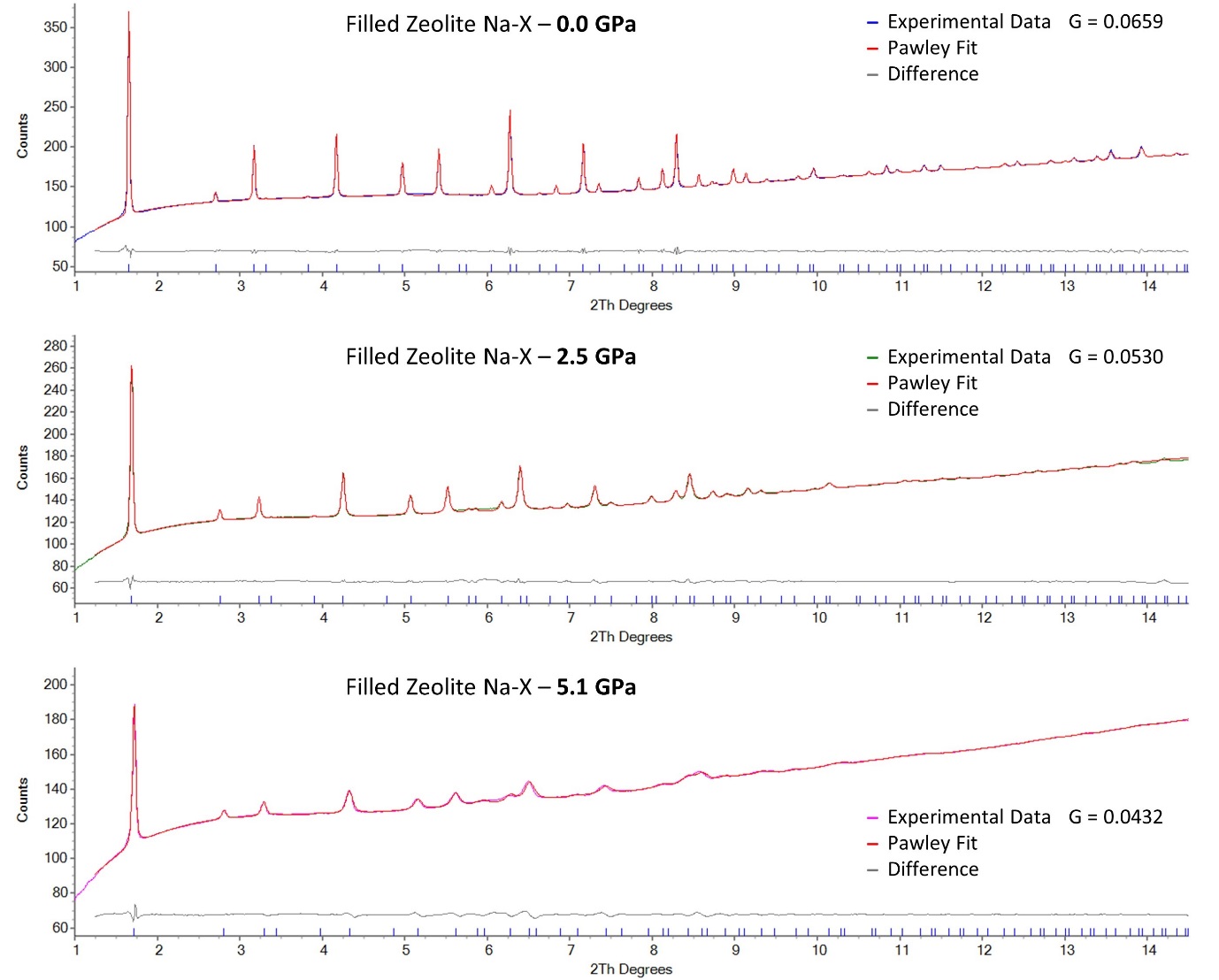
**

**Figure S14.** Final refined Pawley fits for filled zeolite Na-X (FAU) at pressures of 0.0, 2.5 and 5.1 GPa. The patterns in blue, green and pink correspond to the experimental data, red to the Pawley fit and grey to the difference. G refers to the ‘goodness of fit’.

**
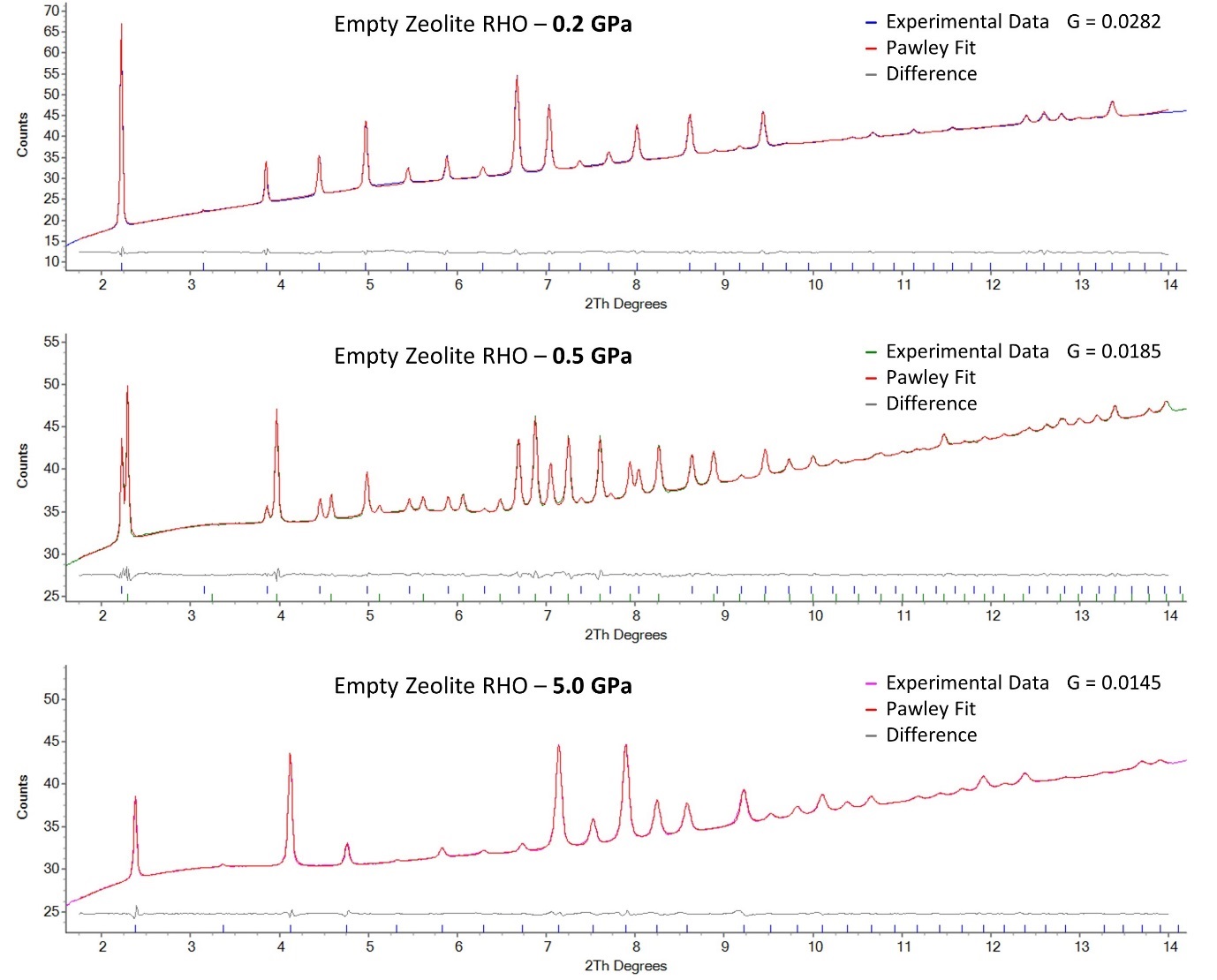
**

**Figure S15.** Final refined Pawley fits for empty zeolite RHO (RHO) at pressures of 0.2, 0.5 and 5.0 GPa. At 0.2 GPa only the C-form is present, at 0.5 GPa both forms are present and at 5.0 GPa only the A-form is present. The patterns in blue, green and pink correspond to the experimental data, red to the Pawley fit and grey to the difference. G refers to the ‘goodness of fit’.

**
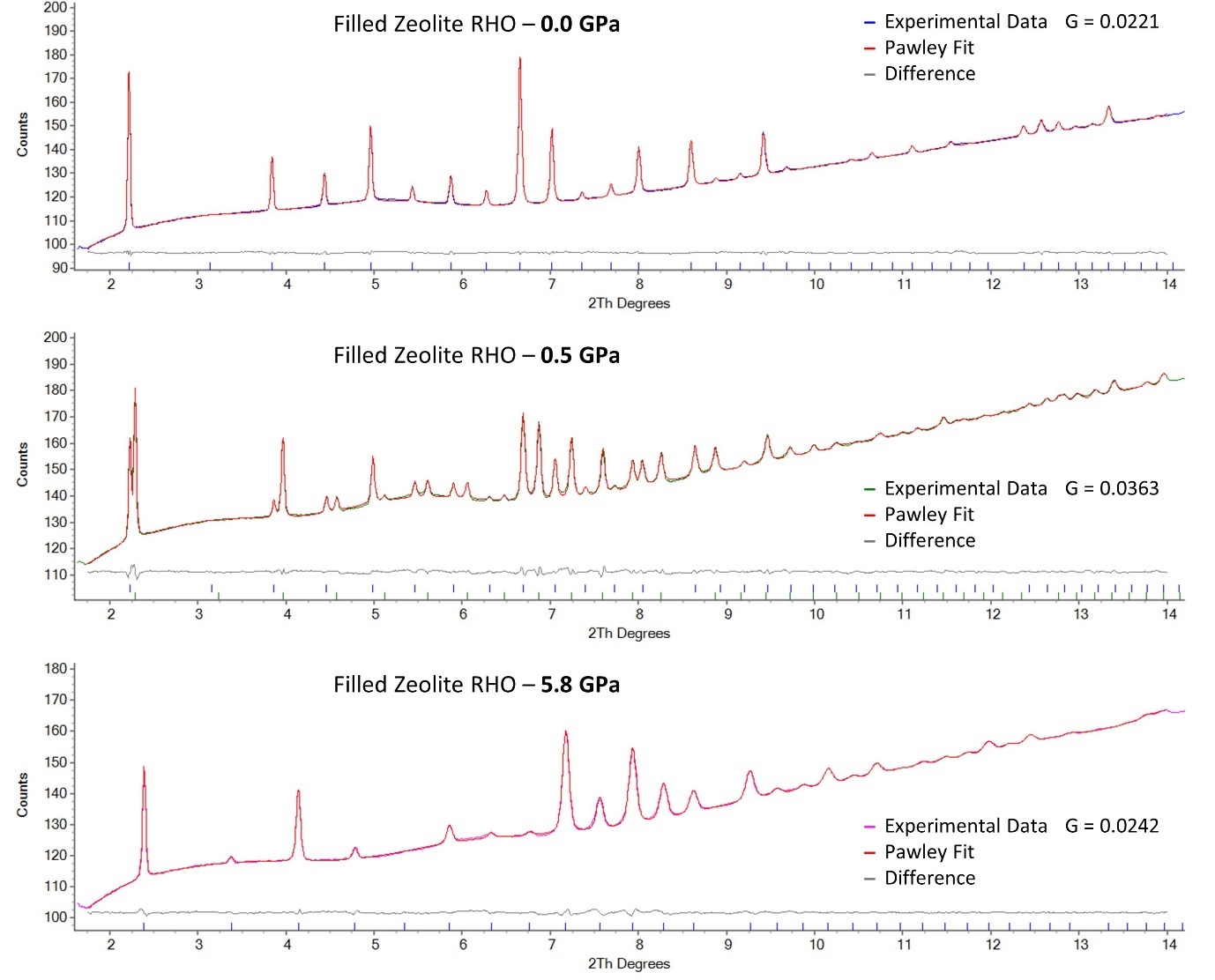
**

**Figure S16.** Final refined Pawley fits for filled zeolite RHO (RHO) at pressures of 0.0, 0.5 and 5.8 GPa. At 0.0 GPa only the C-form is present, at 0.5 GPa both forms are present and at 5.8 GPa only the A-form is present. The patterns in blue, green and pink correspond to the experimental data, red to the Pawley fit and grey to the difference. G refers to the ‘goodness of fit’.

**
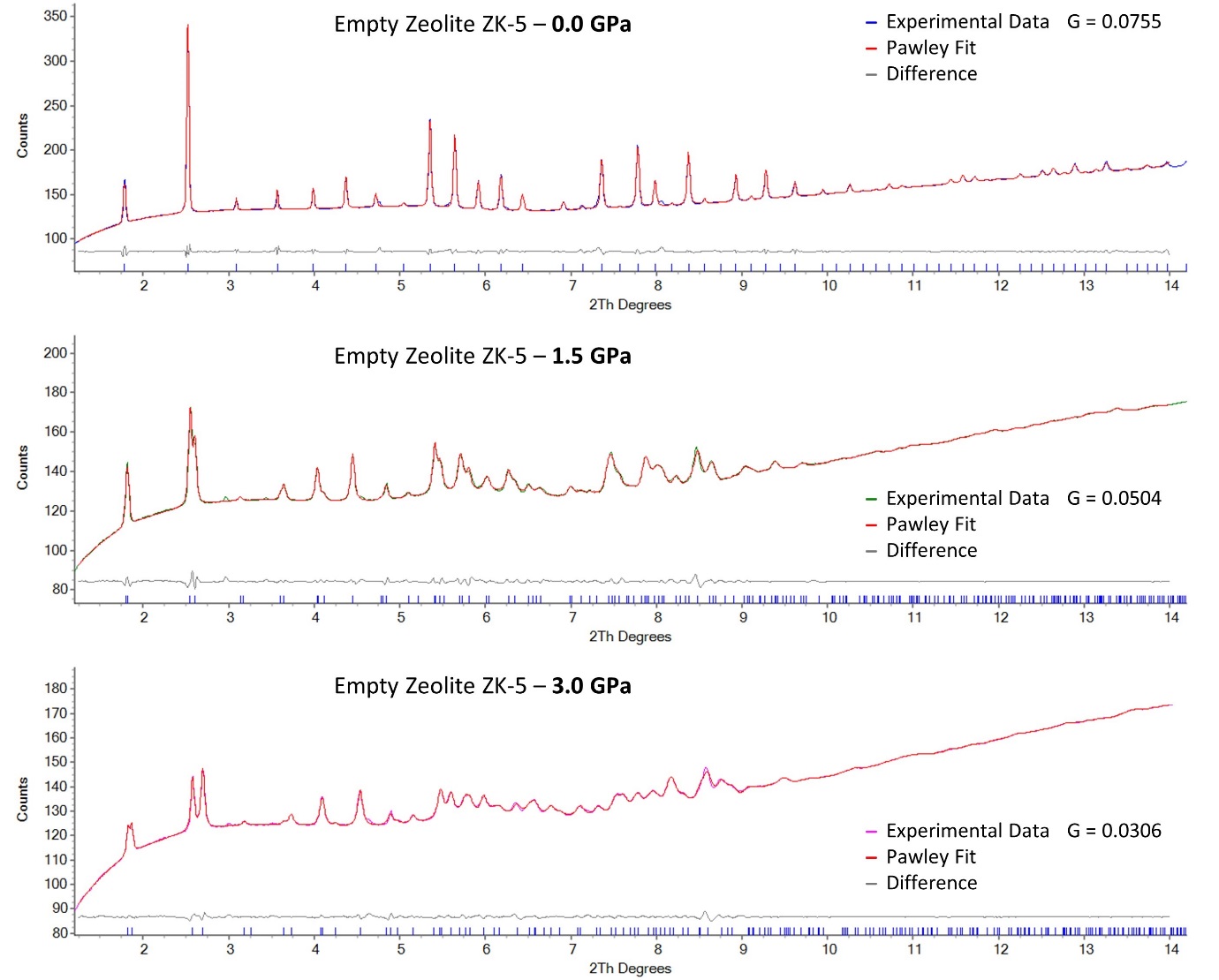
**

**Figure S17.** Final refined Pawley fits for empty zeolite ZK-5 (KFI) at pressures of 0.0, 1.5 and 3.0 GPa. At 0.0 GPa the zeolite is in the cubic phase, and at 1.5 and 3.0 GPa it is in the tetragonal phase. The patterns in blue, green and pink correspond to the experimental data, red to the Pawley fit and grey to the difference. G refers to the ‘goodness of fit’.

**
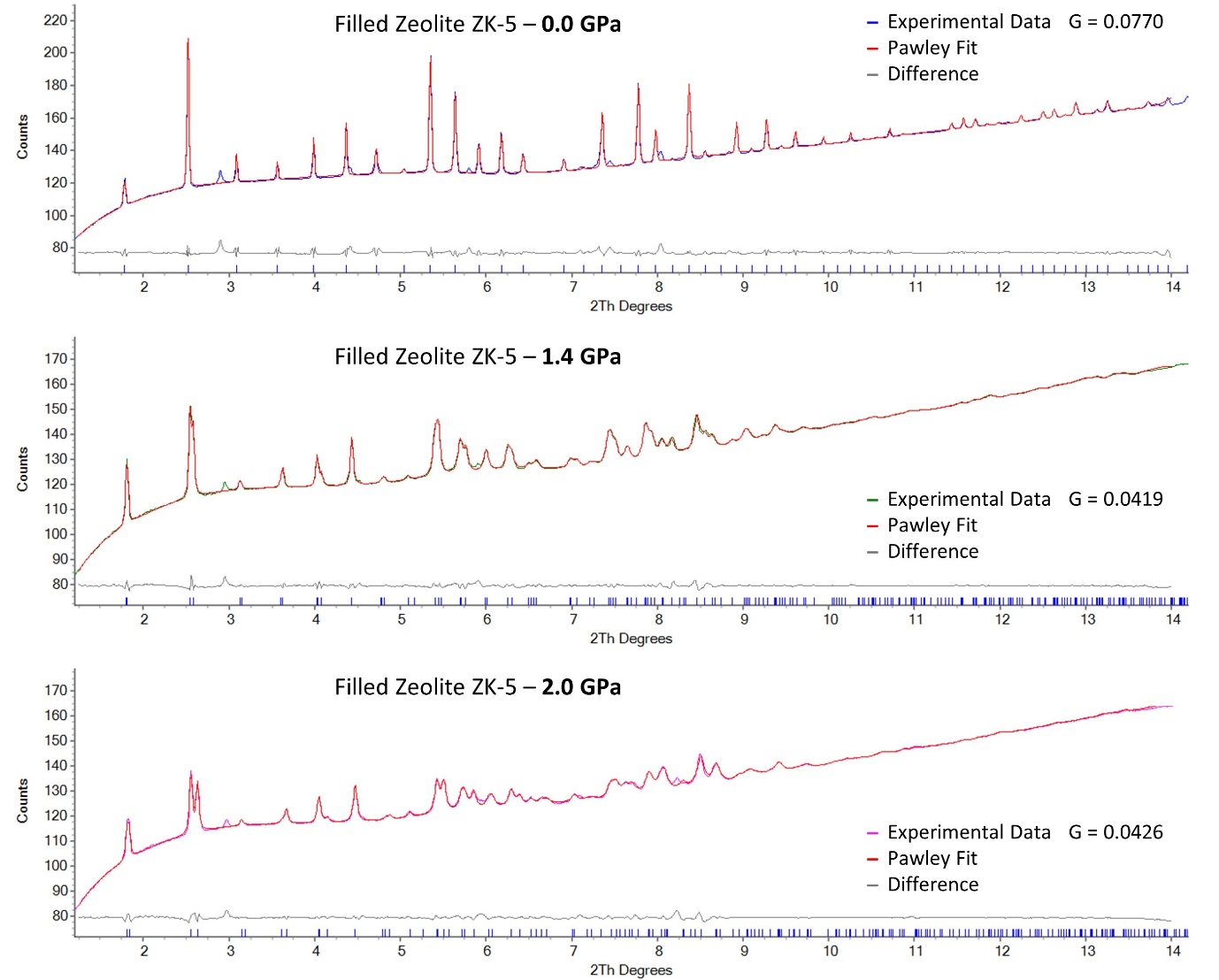
**

**Figure S18.** Final refined Pawley fits for filled zeolite ZK-5 (KFI) at pressures of 0.0, 1.4 and 2.0 GPa. At 0.0 GPa the zeolite is in the cubic phase, and at 1.4 and 2.0 GPa it is in the tetragonal phase. The patterns in blue, green and pink correspond to the experimental data, red to the Pawley fit and grey to the difference. G refers to the ‘goodness of fit’.

**Table S1.** A list of the calculated unit cell parameters and cell volumes for empty zeolite Na-X (FAU). Also included are the errors in these values.

| **Average Pressure /GPa** | **Error /GPa** | **Unit Cell Parameter *a* /Å** | **Error / Å** | **Unit Cell Volume /Å^3^** | **Error / Å^3^** |
| --- | --- | --- | --- | --- | --- |
| 0 | 0.1 | 24.61546 | 1.55E-4 | 14915.01551 | 0.162670229 |
| 0.06 | 0.1 | 24.6029 | 1.69E-4 | 14892.20514 | 0.17718218 |
| 0.095 | 0.1 | 24.57952 | 1.71E-4 | 14849.7841 | 0.178938371 |
| 0.215 | 0.1 | 24.56668 | 1.79E-4 | 14826.51705 | 0.187114021 |
| 0.31 | 0.1 | 24.54851 | 1.81E-4 | 14793.64884 | 0.188924974 |
| 0.41 | 0.1 | 24.5331 | 1.79E-4 | 14765.814 | 0.186603 |
| 0.505 | 0.1 | 24.5199 | 1.84E-4 | 14741.99625 | 0.191609065 |
| 0.635 | 0.1 | 24.49657 | 1.89E-4 | 14699.95109 | 0.19644141 |
| 0.75 | 0.1 | 24.47621 | 1.93E-4 | 14663.32315 | 0.200265515 |
| 0.845 | 0.1 | 24.46217 | 1.99E-4 | 14638.10596 | 0.206254584 |
| 0.95 | 0.1 | 24.43613 | 2E-4 | 14591.40888 | 0.206849952 |
| 1.07 | 0.1 | 24.41326 | 2.08E-4 | 14550.48387 | 0.214721544 |
| 1.165 | 0.1 | 24.39132 | 2.18E-4 | 14511.28634 | 0.22464033 |
| 1.25 | 0.1 | 24.37261 | 2.22E-4 | 14477.91816 | 0.22841135 |
| 1.345 | 0.1 | 24.35316 | 2.3E-4 | 14443.28807 | 0.236264904 |
| 1.44 | 0.1 | 24.33331 | 2.41E-4 | 14407.99737 | 0.24716109 |
| 1.54 | 0.1 | 24.31085 | 2.49E-4 | 14368.12717 | 0.254894224 |
| 1.645 | 0.1 | 24.28884 | 2.55E-4 | 14329.14293 | 0.260563902 |
| 1.755 | 0.1 | 24.26565 | 2.58E-4 | 14288.14651 | 0.263126324 |
| 1.845 | 0.1 | 24.2485 | 2.67E-4 | 14257.86245 | 0.271920175 |
| 1.925 | 0.1 | 24.23108 | 2.57E-4 | 14227.16499 | 0.261360136 |
| 2.04 | 0.1 | 24.21041 | 2.82E-4 | 14190.78365 | 0.286295136 |
| 2.12 | 0.1 | 24.19563 | 2.78E-4 | 14164.80638 | 0.28188965 |
| 2.36 | 0.1 | 24.15888 | 2.93E-4 | 14100.35913 | 0.296197661 |
| 2.76 | 0.1 | 24.11851 | 4.02E-4 | 14029.79641 | 0.405030357 |
| 3.01 | 0.4 | 24.09981 | 5.67E-4 | 13997.18646 | 0.570388572 |
| 3.255 | 0.4 | 24.09098 | 8.47E-4 | 13981.80146 | 0.851437358 |
| 3.49 | 0.4 | 24.07011 | 0.00108 | 13945.50955 | 1.083778909 |
| 3.725 | 0.4 | 24.03638 | 0.00145 | 13886.96691 | 1.450998518 |
| 3.52 | 0.4 | 24.03656 | 0.00171 | 13887.27197 | 1.711202338 |
| 3.065 | 0.4 | 24.06788 | 0.00187 | 13941.62871 | 1.876194717 |
| 2.76 | 0.4 | 24.10002 | 0.00192 | 13997.55585 | 1.931508671 |
| 2.385 | 0.4 | 24.17439 | 0.002 | 14127.54088 | 2.024424905 |
| 1.965 | 0.4 | 24.2768 | 0.00207 | 14307.84812 | 2.113069852 |
| 1.065 | 0.4 | 24.42951 | 0.00215 | 14579.55499 | 2.222432603 |
| 0.515 | 0.4 | 24.54056 | 0.00191 | 14779.2844 | 1.992337605 |

**Table S2.** A list of the calculated unit cell parameters and cell volumes for filled zeolite Na-X (FAU). Also included are the errors in these values.

| **Average Pressure /GPa** | **Error /GPa** | **Unit Cell Parameter *a* /Å** | **Error / Å** | **Unit Cell Volume /Å^3^** | **Error / Å^3^** |
| --- | --- | --- | --- | --- | --- |
| 0 | 0.1 | 24.63234 | 1.99E-4 | 14945.7313 | 0.209134 |
| 0.225 | 0.1 | 24.58569 | 1.94E-4 | 14860.96434 | 0.203108 |
| 0.39 | 0.1 | 24.56951 | 1.99E-4 | 14831.64878 | 0.208069 |
| 0.495 | 0.1 | 24.52016 | 2.15E-4 | 14742.46161 | 0.223896 |
| 0.6 | 0.1 | 24.50054 | 2.29E-4 | 14707.09923 | 0.238093 |
| 0.71 | 0.1 | 24.4769 | 2.34E-4 | 14664.55969 | 0.242823 |
| 0.81 | 0.1 | 24.45578 | 2.43E-4 | 14626.63588 | 0.251727 |
| 0.91 | 0.1 | 24.43431 | 2.49E-4 | 14588.15599 | 0.25749 |
| 1.005 | 0.1 | 24.4136 | 2.55E-4 | 14551.08823 | 0.263248 |
| 1.105 | 0.1 | 24.39141 | 2.67E-4 | 14511.45233 | 0.275135 |
| 1.215 | 0.1 | 24.36718 | 2.71E-4 | 14468.24367 | 0.278702 |
| 1.315 | 0.1 | 24.34744 | 2.77E-4 | 14433.10793 | 0.284411 |
| 1.41 | 0.1 | 24.32875 | 2.84E-4 | 14399.89349 | 0.291151 |
| 1.525 | 0.1 | 24.3054 | 2.92E-4 | 14358.47684 | 0.298778 |
| 1.605 | 0.1 | 24.29073 | 3.02E-4 | 14332.49351 | 0.308638 |
| 1.72 | 0.1 | 24.26857 | 3.13E-4 | 14293.31052 | 0.319296 |
| 1.805 | 0.1 | 24.25524 | 3.17E-4 | 14269.77079 | 0.323021 |
| 1.91 | 0.1 | 24.23683 | 3.21E-4 | 14237.29742 | 0.326601 |
| 2.02 | 0.1 | 24.23683 | 3.2E-4 | 14237.29037 | 0.325583 |
| 2.515 | 0.1 | 24.15356 | 4.53E-4 | 14091.05136 | 0.457742 |
| 2.755 | 0.1 | 24.13414 | 4.92E-4 | 14057.09886 | 0.496352 |
| 2.995 | 0.1 | 24.10584 | 5.29E-4 | 14007.701 | 0.532428 |
| 3.265 | 0.4 | 24.06496 | 6.32E-4 | 13936.55498 | 0.63394 |
| 3.51 | 0.4 | 24.03377 | 6.76E-4 | 13882.43671 | 0.676318 |
| 3.76 | 0.4 | 23.99344 | 7.46E-4 | 13812.65878 | 0.743848 |
| 4.01 | 0.4 | 23.95081 | 8.07E-4 | 13739.1755 | 0.801816 |
| 4.295 | 0.4 | 23.91064 | 8.73E-4 | 13670.16014 | 0.864485 |
| 4.57 | 0.4 | 23.85457 | 9.75E-4 | 13574.20985 | 0.960966 |
| 4.75 | 0.4 | 23.80106 | 0.00103 | 13483.08016 | 1.010626 |
| 4.895 | 0.4 | 23.76622 | 0.00108 | 13423.95041 | 1.056585 |
| 5.05 | 0.4 | 23.73155 | 0.00113 | 13365.28776 | 1.102278 |
| 4.76 | 0.4 | 23.71757 | 0.00112 | 13997.55585 | 1.144882 |
| 4.525 | 0.4 | 23.72061 | 0.00117 | 14127.54088 | 1.206944 |
| 4.015 | 0.4 | 23.74352 | 0.00119 | 14307.84812 | 1.242043 |
| 3.505 | 0.4 | 23.78691 | 0.0012 | 14579.55499 | 1.273937 |
| 2.915 | 0.4 | 23.85945 | 0.00118 | 13582.5491 | 1.163492 |
| 1.39 | 0.4 | 24.24434 | 0.00125 | 14250.5327 | 1.272598 |
| 1.09 | 0.4 | 24.30911 | 0.00123 | 14365.0511 | 1.258937 |
| 0.535 | 0.4 | 24.4438 | 0.00108 | 14605.1548 | 1.117691 |

**Table S3.** A list of the calculated unit cell parameters for empty zeolite RHO. Also included are the errors in these values.

| **Average Pressure /GPa** | **Error /GPa** | **Im-3m Unit Cell Parameter *a* /Å** | **Error / Å** | **I-43m Unit Cell Parameter *a* /Å** | **Error / Å** |
| --- | --- | --- | --- | --- | --- |
| 0.16 | 0.1 | 14.99421 | 1.26E-4 |  |  |
| 0.275 | 0.1 | 14.97595 | 1E-4 | 14.55773 | 0.00109 |
| 0.365 | 0.1 | 14.9697 | 1.35E-4 | 14.5591 | 2.81E-4 |
| 0.5 | 0.1 | 14.95591 | 2.69E-4 | 14.54617 | 1.61E-4 |
| 0.595 | 0.1 | 14.93907 | 3.56E-4 | 14.52714 | 1.29E-4 |
| 0.71 | 0.1 | 14.91823 | 6.52E-4 | 14.50469 | 1.17E-4 |
| 0.845 | 0.1 | 14.90669 | 0.00168 | 14.47799 | 1.05E-4 |
| 0.96 | 0.1 | 14.8951 | 0.00352 | 14.45002 | 1.03E-4 |
| 1.095 | 0.1 |  |  | 14.4209 | 1E-4 |
| 1.28 | 0.1 |  |  | 14.39237 | 9.6E-5 |
| 1.385 | 0.1 |  |  | 14.3622 | 9.8E-5 |
| 1.515 | 0.1 |  |  | 14.33987 | 9E-5 |
| 1.655 | 0.1 |  |  | 14.31702 | 9E-5 |
| 1.795 | 0.1 |  |  | 14.29563 | 9.2E-5 |
| 1.935 | 0.1 |  |  | 14.27355 | 9.5E-5 |
| 2.075 | 0.1 |  |  | 14.25322 | 9.2E-5 |
| 2.21 | 0.1 |  |  | 14.23524 | 9.2E-5 |
| 2.335 | 0.1 |  |  | 14.22118 | 9.1E-5 |
| 2.61 | 0.1 |  |  | 14.20073 | 9.7E-5 |
| 2.905 | 0.1 |  |  | 14.18151 | 1E-4 |
| 3.215 | 0.4 |  |  | 14.1485 | 1.01E-4 |
| 3.525 | 0.4 |  |  | 14.12624 | 1.05E-4 |
| 3.865 | 0.4 |  |  | 14.10141 | 1.1E-4 |
| 4.195 | 0.4 |  |  | 14.08337 | 1.18E-4 |
| 4.51 | 0.4 |  |  | 14.07692 | 1.2E-4 |
| 4.84 | 0.4 |  |  | 14.03134 | 1.38E-4 |
| 5.045 | 0.4 |  |  | 14.01 | 1.37E-4 |
| 4.055 | 0.4 |  |  | 14.04469 | 1.34E-4 |
| 3.095 | 0.4 |  |  | 14.12174 | 1.33E-4 |
| 1.895 | 0.4 |  |  | 14.28701 | 1.18E-4 |
| 1.22 | 0.4 |  |  | 14.38347 | 1.22E-4 |
| 0.615 | 0.4 | 14.93104 | 0.00224 | 14.52046 | 1.16E-4 |
| 0.135 | 0.4 | 14.97409 | 2.66E-4 | 14.61383 | 7.63E-4 |
| 0.015 | 0.4 | 14.9911 | 1.96E-4 |  |  |

**Table S4.** A list of the calculated unit cell volumes for empty zeolite RHO. Also included are the errors in these values.

| **Average Pressure /GPa** | **Error /GPa** | **Im-3m Unit Cell Volume /Å^3^** | **Error / Å^3^** | **I-43m Unit Cell Volume /Å^3^** | **Error / Å^3^** |
| --- | --- | --- | --- | --- | --- |
| 0.16 | 0.1 | 3371.09326 | 0.049066 |  |  |
| 0.275 | 0.1 | 3358.79428 | 0.038846 | 3085.18146 | 0.400105 |
| 0.365 | 0.1 | 3354.59215 | 0.052399 | 3086.05193 | 0.103166 |
| 0.5 | 0.1 | 3345.32933 | 0.104217 | 3077.83892 | 0.059004 |
| 0.595 | 0.1 | 3334.03975 | 0.137612 | 3065.77498 | 0.047153 |
| 0.71 | 0.1 | 3320.10292 | 0.251329 | 3051.58165 | 0.042635 |
| 0.845 | 0.1 | 3312.40874 | 0.646595 | 3034.76137 | 0.038121 |
| 0.96 | 0.1 | 3304.6832 | 1.352663 | 3017.20928 | 0.037251 |
| 1.095 | 0.1 |  |  | 2999.00123 | 0.036020 |
| 1.28 | 0.1 |  |  | 2981.23756 | 0.034443 |
| 1.385 | 0.1 |  |  | 2962.52919 | 0.035013 |
| 1.515 | 0.1 |  |  | 2948.73369 | 0.032055 |
| 1.655 | 0.1 |  |  | 2934.65823 | 0.031953 |
| 1.795 | 0.1 |  |  | 2921.52634 | 0.032565 |
| 1.935 | 0.1 |  |  | 2908.01194 | 0.033523 |
| 2.075 | 0.1 |  |  | 2895.60021 | 0.032372 |
| 2.21 | 0.1 |  |  | 2884.65711 | 0.032291 |
| 2.335 | 0.1 |  |  | 2876.11933 | 0.031877 |
| 2.61 | 0.1 |  |  | 2863.72719 | 0.033881 |
| 2.905 | 0.1 |  |  | 2852.1194 | 0.034834 |
| 3.215 | 0.4 |  |  | 2832.24687 | 0.035019 |
| 3.525 | 0.4 |  |  | 2818.90226 | 0.036291 |
| 3.865 | 0.4 |  |  | 2804.06086 | 0.037886 |
| 4.195 | 0.4 |  |  | 2793.31109 | 0.040537 |
| 4.51 | 0.4 |  |  | 2789.47851 | 0.041187 |
| 4.84 | 0.4 |  |  | 2762.46625 | 0.047058 |
| 5.045 | 0.4 |  |  | 2749.88656 | 0.046576 |
| 4.055 | 0.4 |  |  | 2770.36169 | 0.045781 |
| 3.095 | 0.4 |  |  | 2816.20739 | 0.045940 |
| 1.895 | 0.4 |  |  | 2916.24526 | 0.041718 |
| 1.22 | 0.4 |  |  | 2975.71282 | 0.043717 |
| 0.615 | 0.4 | 3328.66567 | 0.864946 | 3061.54836 | 0.042362 |
| 0.135 | 0.4 | 3357.54094 | 0.103305 | 3120.98839 | 0.282237 |
| 0.015 | 0.4 | 3368.99606 | 0.076293 |  |  |

**Table S5.** A list of the calculated unit cell parameters for filled zeolite RHO. Also included are the errors in these values.

| **Average Pressure /GPa** | **Error /GPa** | **Im-3m Unit Cell Parameter *a* /Å** | **Error / Å** | **I-43m Unit Cell Parameter *a* /Å** | **Error / Å** |
| --- | --- | --- | --- | --- | --- |
| 0 | 0.1 | 15.02405 | 8.1E-5 |  |  |
| 0.09 | 0.1 | 15.01513 | 7.2E-5 |  |  |
| 0.17 | 0.1 | 15.00294 | 7.5E-5 |  |  |
| 0.245 | 0.1 | 14.98472 | 8E-5 |  |  |
| 0.33 | 0.1 | 14.9713 | 1.17E-4 | 14.60415 | 6.13E-4 |
| 0.365 | 0.1 | 14.95515 | 1.86E-4 | 14.56774 | 3.11E-4 |
| 0.485 | 0.1 | 14.94541 | 3.04E-4 | 14.56059 | 2.43E-4 |
| 0.825 | 0.1 | 14.89554 | 0.00101 | 14.50228 | 1.37E-4 |
| 0.985 | 0.1 | 14.86799 | 0.00184 | 14.46847 | 1.38E-4 |
| 1.115 | 0.1 | 14.85293 | 0.00266 | 14.44455 | 1.4E-4 |
| 1.25 | 0.1 |  |  | 14.4183 | 1.51E-4 |
| 1.39 | 0.1 |  |  | 14.39366 | 9.6E-5 |
| 1.535 | 0.1 |  |  | 14.36772 | 1.44E-4 |
| 1.685 | 0.1 |  |  | 14.34381 | 1.43E-4 |
| 1.83 | 0.1 |  |  | 14.32428 | 1.43E-4 |
| 2.03 | 0.1 |  |  | 14.29733 | 1.42E-4 |
| 2.23 | 0.1 |  |  | 14.27186 | 1.36E-4 |
| 2.455 | 0.1 |  |  | 14.24795 | 1.34E-4 |
| 2.67 | 0.1 |  |  | 14.22871 | 1.35E-4 |
| 2.905 | 0.1 |  |  | 14.20396 | 1.35E-4 |
| 3.225 | 0.4 |  |  | 14.17224 | 1.33E-4 |
| 3.54 | 0.4 |  |  | 14.1378 | 1.32E-4 |
| 3.825 | 0.4 |  |  | 14.10197 | 1.4E-4 |
| 4.175 | 0.4 |  |  | 14.07199 | 1.55E-4 |
| 4.505 | 0.4 |  |  | 14.04468 | 1.64E-4 |
| 4.93 | 0.4 |  |  | 14.00662 | 1.7E-4 |
| 5.16 | 0.4 |  |  | 13.99254 | 1.85E-4 |
| 5.515 | 0.4 |  |  | 13.96113 | 1.89E-4 |
| 5.76 | 0.4 |  |  | 13.94082 | 2.22E-4 |
| 5.18 | 0.4 |  |  | 13.96414 | 2.01E-4 |
| 4.66 | 0.4 |  |  | 13.99864 | 1.66E-4 |
| 3.965 | 0.4 |  |  | 14.05472 | 1.69E-4 |
| 3.505 | 0.4 |  |  | 14.0962 | 1.58E-4 |
| 3.015 | 0.4 |  |  | 14.16015 | 1.43E-4 |
| 2.435 | 0.4 |  |  | 14.25078 | 1.5E-4 |
| 1.965 | 0.4 |  |  | 14.32871 | 1.28E-4 |
| 1.475 | 0.4 |  |  | 14.40929 | 1.23E-4 |
| 0.285 | 0.4 | 14.96276 | 2.47E-4 | 14.66969 | 0.00139 |
| 0.1 | 0.4 | 14.98792 | 1.9E-4 |  |  |

**Table S6.** A list of the calculated unit cell volumes for filled zeolite RHO. Also included are the errors in these values.

| **Average Pressure /GPa** | **Error /GPa** | **Im-3m Unit Cell Volume /Å^3^** | **Error / Å^3^** | **I-43m Unit Cell Volume /Å^3^** | **Error / Å^3^** |
| --- | --- | --- | --- | --- | --- |
| 0 | 0.1 | 3391.26047 | 0.031668 |  |  |
| 0.09 | 0.1 | 3385.22035 | 0.028116 |  |  |
| 0.17 | 0.1 | 3376.98624 | 0.029240 |  |  |
| 0.245 | 0.1 | 3364.69583 | 0.031113 |  |  |
| 0.33 | 0.1 | 3355.66723 | 0.045422 | 3114.79124 | 0.226451 |
| 0.365 | 0.1 | 3344.81601 | 0.072053 | 3091.55383 | 0.114316 |
| 0.485 | 0.1 | 3338.28502 | 0.117612 | 3087.00333 | 0.089233 |
| 0.825 | 0.1 | 3304.98139 | 0.388146 | 3050.06586 | 0.049906 |
| 0.985 | 0.1 | 3286.6725 | 0.704503 | 3028.78318 | 0.050036 |
| 1.115 | 0.1 | 3276.69658 | 1.016404 | 3013.78474 | 0.050594 |
| 1.25 | 0.1 |  |  | 2997.38129 | 0.054371 |
| 1.39 | 0.1 |  |  | 2982.04175 | 0.034449 |
| 1.535 | 0.1 |  |  | 2965.94514 | 0.051487 |
| 1.685 | 0.1 |  |  | 2951.16801 | 0.050960 |
| 1.83 | 0.1 |  |  | 2939.12797 | 0.050821 |
| 2.03 | 0.1 |  |  | 2922.56995 | 0.050276 |
| 2.23 | 0.1 |  |  | 2906.97729 | 0.047980 |
| 2.455 | 0.1 |  |  | 2892.38953 | 0.047116 |
| 2.67 | 0.1 |  |  | 2880.69161 | 0.047340 |
| 2.905 | 0.1 |  |  | 2865.68415 | 0.047175 |
| 3.225 | 0.4 |  |  | 2846.52823 | 0.046269 |
| 3.54 | 0.4 |  |  | 2825.82655 | 0.045698 |
| 3.825 | 0.4 |  |  | 2804.39613 | 0.048222 |
| 4.175 | 0.4 |  |  | 2786.54519 | 0.053162 |
| 4.505 | 0.4 |  |  | 2770.35341 | 0.056031 |
| 4.93 | 0.4 |  |  | 2747.8944 | 0.057767 |
| 5.16 | 0.4 |  |  | 2739.61527 | 0.062737 |
| 5.515 | 0.4 |  |  | 2721.20784 | 0.063806 |
| 5.76 | 0.4 |  |  | 2709.34613 | 0.074729 |
| 5.18 | 0.4 |  |  | 2722.96828 | 0.067887 |
| 4.66 | 0.4 |  |  | 2743.20040 | 0.056343 |
| 3.965 | 0.4 |  |  | 2776.30128 | 0.057822 |
| 3.505 | 0.4 |  |  | 2800.95518 | 0.054378 |
| 3.015 | 0.4 |  |  | 2839.24952 | 0.049663 |
| 2.435 | 0.4 |  |  | 2894.11582 | 0.052763 |
| 1.965 | 0.4 |  |  | 2941.85511 | 0.045518 |
| 1.475 | 0.4 |  |  | 2991.76685 | 0.044233 |
| 0.285 | 0.4 | 3349.92536 | 0.095781 | 3156.91442 | 0.518104 |
| 0.1 | 0.4 | 3366.85256 | 0.073926 |  |  |

**Table S7.** A list of the calculated unit cell parameters for empty zeolite ZK-5. Also included are the errors in these values.

| **Average Pressure /GPa** | **Error /GPa** | **Unit Cell Parameter *a* /Å** | **Error / Å** | **Tetragonal Unit Cell Parameter *c* /Å** | **Error / Å** |
| --- | --- | --- | --- | --- | --- |
| 0 | 0.1 | 18.67993 | 1.61E-4 |  |  |
| 0.04 | 0.1 | 18.67618 | 1.65E-4 |  |  |
| 0.15 | 0.1 | 18.66103 | 2.41E-4 |  |  |
| 0.29 | 0.1 | 18.64393 | 2.38E-4 |  |  |
| 0.4 | 0.1 | 18.63198 | 2.41E-4 |  |  |
| 0.505 | 0.1 | 18.61926 | 2.45E-4 |  |  |
| 0.605 | 0.1 | 18.60678 | 2.18E-4 |  |  |
| 0.71 | 0.1 | 18.59468 | 2.3E-4 |  |  |
| 0.81 | 0.1 | 18.58047 | 2.24E-4 |  |  |
| 0.91 | 0.1 | 18.56526 | 1.84E-4 |  |  |
| 1.02 | 0.1 | 18.54869 | 2.1E-4 |  |  |
| 1.13 | 0.1 | 18.53066 | 2.19E-4 |  |  |
| 1.225 | 0.1 | 18.51399 | 2.55E-4 |  |  |
| 1.34 | 0.1 | 18.4882 | 4.4E-4 |  |  |
| 1.52 | 0.1 | 18.48196 | 0.00102 | 18.08293 | 0.00155 |
| 1.625 | 0.1 | 18.46559 | 0.00112 | 18.00553 | 0.00148 |
| 1.83 | 0.1 | 18.43305 | 0.00116 | 17.84002 | 0.00135 |
| 1.915 | 0.1 | 18.42271 | 0.00104 | 17.79639 | 0.00123 |
| 2.005 | 0.1 | 18.41064 | 0.00104 | 17.7589 | 0.0012 |
| 2.115 | 0.1 | 18.39201 | 0.00105 | 17.70605 | 0.00116 |
| 2.205 | 0.1 | 18.37934 | 0.00104 | 17.67133 | 0.00113 |
| 2.305 | 0.1 | 18.34417 | 0.00102 | 17.5955 | 0.00107 |
| 2.415 | 0.1 | 18.34391 | 0.00102 | 17.59518 | 0.00107 |
| 2.615 | 0.1 | 18.32272 | 0.00107 | 17.55102 | 0.00111 |
| 3.02 | 0.4 | 18.27538 | 0.00122 | 17.46641 | 0.00122 |
| 2.585 | 0.4 | 18.30089 | 0.0012 | 17.53364 | 0.00123 |
| 2.34 | 0.4 | 18.32949 | 0.00118 | 17.60025 | 0.00124 |
| 1.42 | 0.4 | 18.48979 | 0.00117 | 18.02056 | 0.0015 |
| 1.055 | 0.4 | 18.52761 | 0.00108 | 18.20229 | 0.00203 |
| 0.825 | 0.4 | 18.55714 | 0.0013 | 18.36098 | 0.00326 |
| 0.63 | 0.4 | 18.57326 | 4.98E-4 |  |  |

**Table S8.** A list of the calculated unit cell volumes for empty zeolite ZK-5. Also included are the errors in these values.

| **Average Pressure /GPa** | **Error /GPa** | **Cubic Unit Cell Volume /Å^3^** | **Error / Å^3^** | **Tetragonal Unit Cell Volume /Å^3^** | **Error / Å^3^** |
| --- | --- | --- | --- | --- | --- |
| 0 | 0.1 | 6518.16761 | 0.097305 |  |  |
| 0.04 | 0.1 | 6514.24806 | 0.099683 |  |  |
| 0.15 | 0.1 | 6498.40588 | 0.145361 |  |  |
| 0.29 | 0.1 | 6480.55784 | 0.143289 |  |  |
| 0.4 | 0.1 | 6468.10659 | 0.144909 |  |  |
| 0.505 | 0.1 | 6454.86523 | 0.147113 |  |  |
| 0.605 | 0.1 | 6441.89124 | 0.130725 |  |  |
| 0.71 | 0.1 | 6429.33709 | 0.137742 |  |  |
| 0.81 | 0.1 | 6414.60852 | 0.133944 |  |  |
| 0.91 | 0.1 | 6398.86218 | 0.109845 |  |  |
| 1.02 | 0.1 | 6381.75431 | 0.125143 |  |  |
| 1.13 | 0.1 | 6363.15427 | 0.130253 |  |  |
| 1.225 | 0.1 | 6345.99804 | 0.151391 |  |  |
| 1.34 | 0.1 | 6319.51605 | 0.260497 |  |  |
| 1.52 | 0.1 |  |  | 6176.81901 | 0.716056 |
| 1.625 | 0.1 |  |  | 6139.48688 | 0.729386 |
| 1.83 | 0.1 |  |  | 6061.63311 | 0.708118 |
| 1.915 | 0.1 |  |  | 6040.02394 | 0.637804 |
| 2.005 | 0.1 |  |  | 6019.41335 | 0.629827 |
| 2.115 | 0.1 |  |  | 5989.35696 | 0.622739 |
| 2.205 | 0.1 |  |  | 5969.37936 | 0.611470 |
| 2.305 | 0.1 |  |  | 5921.04052 | 0.588585 |
| 2.415 | 0.1 |  |  | 5920.76402 | 0.588566 |
| 2.615 | 0.1 |  |  | 5892.26278 | 0.612919 |
| 3.02 | 0.4 |  |  | 5833.59551 | 0.685085 |
| 2.585 | 0.4 |  |  | 5872.41018 | 0.682821 |
| 2.34 | 0.4 |  |  | 5913.15666 | 0.680720 |
| 1.42 | 0.4 |  |  | 6160.72954 | 0.752942 |
| 1.055 | 0.4 |  |  | 6248.34053 | 0.866550 |
| 0.825 | 0.4 |  |  | 6322.92207 | 1.285580 |
| 0.63 | 0.4 | 6407.14193 | 0.297554 |  |  |

**Table S9.** A list of the calculated unit cell parameters for filled zeolite ZK-5. Also included are the errors in these values.

| **Average Pressure /GPa** | **Error /GPa** | **Unit Cell Parameter *a* /Å** | **Error / Å** | **Tetragonal Unit Cell Parameter *c* /Å** | **Error / Å** |
| --- | --- | --- | --- | --- | --- |
| 0 | 0.1 | 18.69001 | 2.14E-4 |  |  |
| 0.195 | 0.1 | 18.66394 | 2.41E-4 |  |  |
| 0.19 | 0.1 | 18.66465 | 2.26E-4 |  |  |
| 0.3 | 0.1 | 18.65386 | 2.03E-4 |  |  |
| 0.405 | 0.1 | 18.63931 | 1.95E-4 |  |  |
| 0.505 | 0.1 | 18.62453 | 1.91E-4 |  |  |
| 0.615 | 0.1 | 18.60878 | 1.94E-4 |  |  |
| 0.695 | 0.1 | 18.59494 | 2.04E-4 |  |  |
| 0.8 | 0.1 | 18.57772 | 2.19E-4 |  |  |
| 0.895 | 0.1 | 18.56136 | 2.31E-4 |  |  |
| 1.025 | 0.1 | 18.53556 | 2.76E-4 |  |  |
| 1.11 | 0.1 | 18.51515 | 3.69E-4 |  |  |
| 1.22 | 0.1 | 18.4884 | 5.41E-4 |  |  |
| 1.32 | 0.1 | 18.45558 | 9.29E-4 |  |  |
| 1.425 | 0.1 | 18.50978 | 0.00103 | 18.23958 | 0.00139 |
| 1.535 | 0.1 | 18.49816 | 0.00102 | 18.17126 | 0.00141 |
| 1.67 | 0.1 | 18.48103 | 0.0011 | 18.09363 | 0.00153 |
| 1.74 | 0.1 | 18.47181 | 0.00118 | 18.04833 | 0.00163 |
| 1.835 | 0.1 | 18.45797 | 0.00131 | 17.99519 | 0.00179 |
| 1.93 | 0.1 | 18.44518 | 0.00136 | 17.93937 | 0.00187 |
| 2.01 | 0.1 | 18.43514 | 0.00148 | 17.89729 | 0.00194 |
| 1.96 | 0.4 | 18.4409 | 0.00138 | 17.92355 | 0.00185 |
| 1.5 | 0.4 | 18.51198 | 8.6E-4 | 18.18793 | 0.00123 |
| 0.92 | 0.4 | 18.54605 | 3.45E-4 |  |  |
| 0.475 | 0.4 | 18.62175 | 1.99E-4 |  |  |

**Table S10.** A list of the calculated unit cell volumes for filled zeolite ZK-5. Also included are the errors in these values.

| **Average Pressure /GPa** | **Error /GPa** | **Cubic Unit Cell Volume /Å^3^** | **Error / Å^3^** | **Tetragonal Unit Cell Volume /Å^3^** | **Error / Å^3^** |
| --- | --- | --- | --- | --- | --- |
| 0 | 0.1 | 6528.73258 | 0.129477 |  |  |
| 0.195 | 0.1 | 6501.45062 | 0.145407 |  |  |
| 0.19 | 0.1 | 6502.1853 | 0.136367 |  |  |
| 0.3 | 0.1 | 6490.9172 | 0.122347 |  |  |
| 0.405 | 0.1 | 6475.74031 | 0.117342 |  |  |
| 0.505 | 0.1 | 6460.34879 | 0.114753 |  |  |
| 0.615 | 0.1 | 6443.96977 | 0.116358 |  |  |
| 0.695 | 0.1 | 6429.60057 | 0.122174 |  |  |
| 0.8 | 0.1 | 6411.75869 | 0.130915 |  |  |
| 0.895 | 0.1 | 6394.83144 | 0.137845 |  |  |
| 1.025 | 0.1 | 6368.21058 | 0.164241 |  |  |
| 1.11 | 0.1 | 6347.18786 | 0.219099 |  |  |
| 1.22 | 0.1 | 6319.72217 | 0.320300 |  |  |
| 1.32 | 0.1 | 6286.12312 | 0.548064 |  |  |
| 1.425 | 0.1 |  |  | 6249.09986 | 0.684573 |
| 1.535 | 0.1 |  |  | 6217.87334 | 0.684022 |
| 1.67 | 0.1 |  |  | 6179.85234 | 0.737342 |
| 1.74 | 0.1 |  |  | 6158.23065 | 0.786665 |
| 1.835 | 0.1 |  |  | 6130.89771 | 0.866358 |
| 1.93 | 0.1 |  |  | 6103.41744 | 0.899893 |
| 2.01 | 0.1 |  |  | 6082.47352 | 0.954774 |
| 1.96 | 0.4 |  |  | 6095.1995 | 0.901054 |
| 1.5 | 0.4 |  |  | 6232.88701 | 0.587674 |
| 0.92 | 0.4 | 6379.02772 | 0.205534 |  |  |
| 0.475 | 0.4 | 6457.45838 | 0.119524 |  |  |


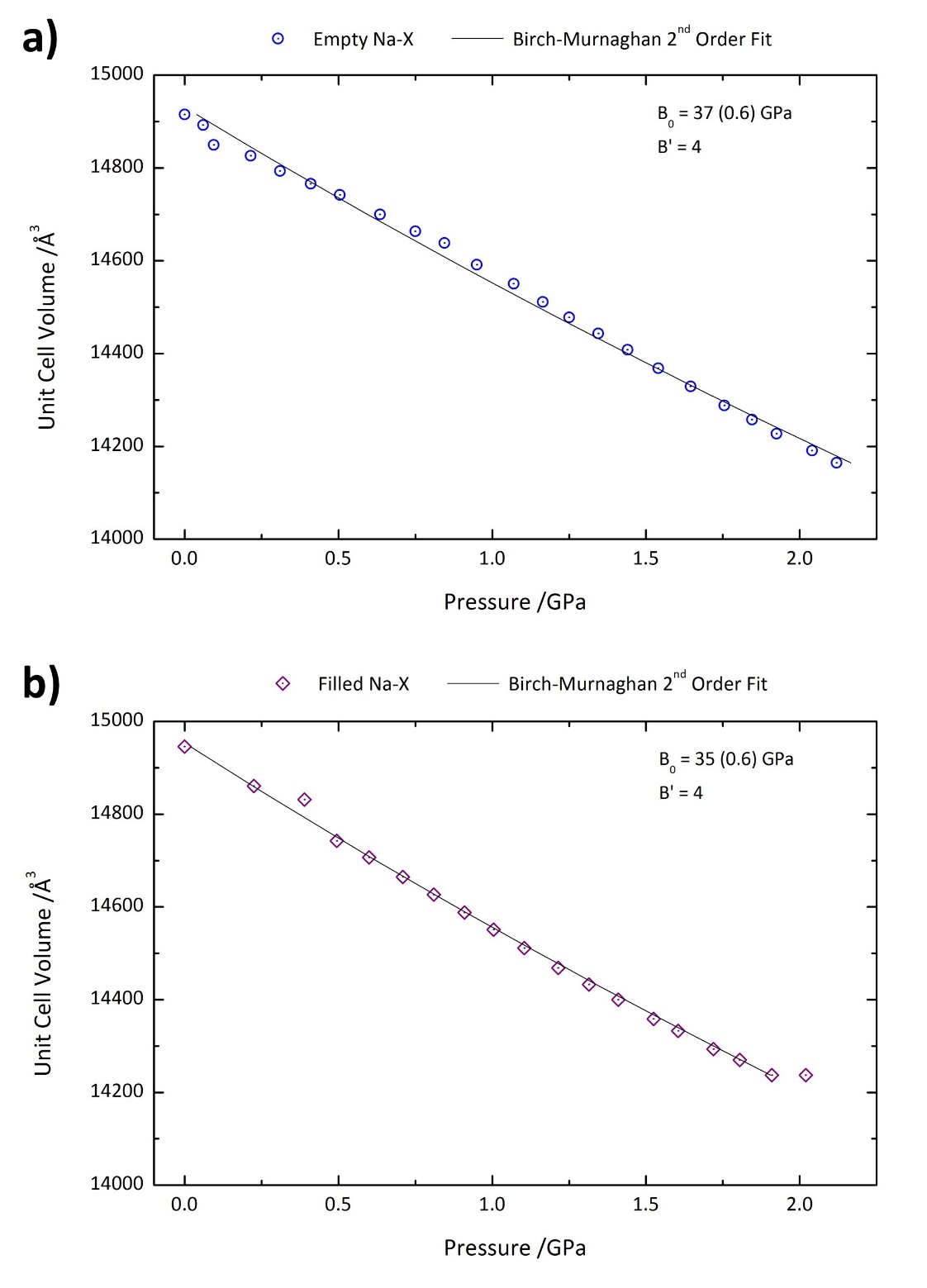


**Figure S19.** The variation in unit cell volume with pressure for a) the empty and b) the filled zeolite Na-X samples, in the 0-2.2 GPa pressure range. Shown are the Birch-Murnaghan 2^nd^ order fits, bulk moduli (B_0_) and pressure derivatives (B’) determined using the webtool PASCal [47].


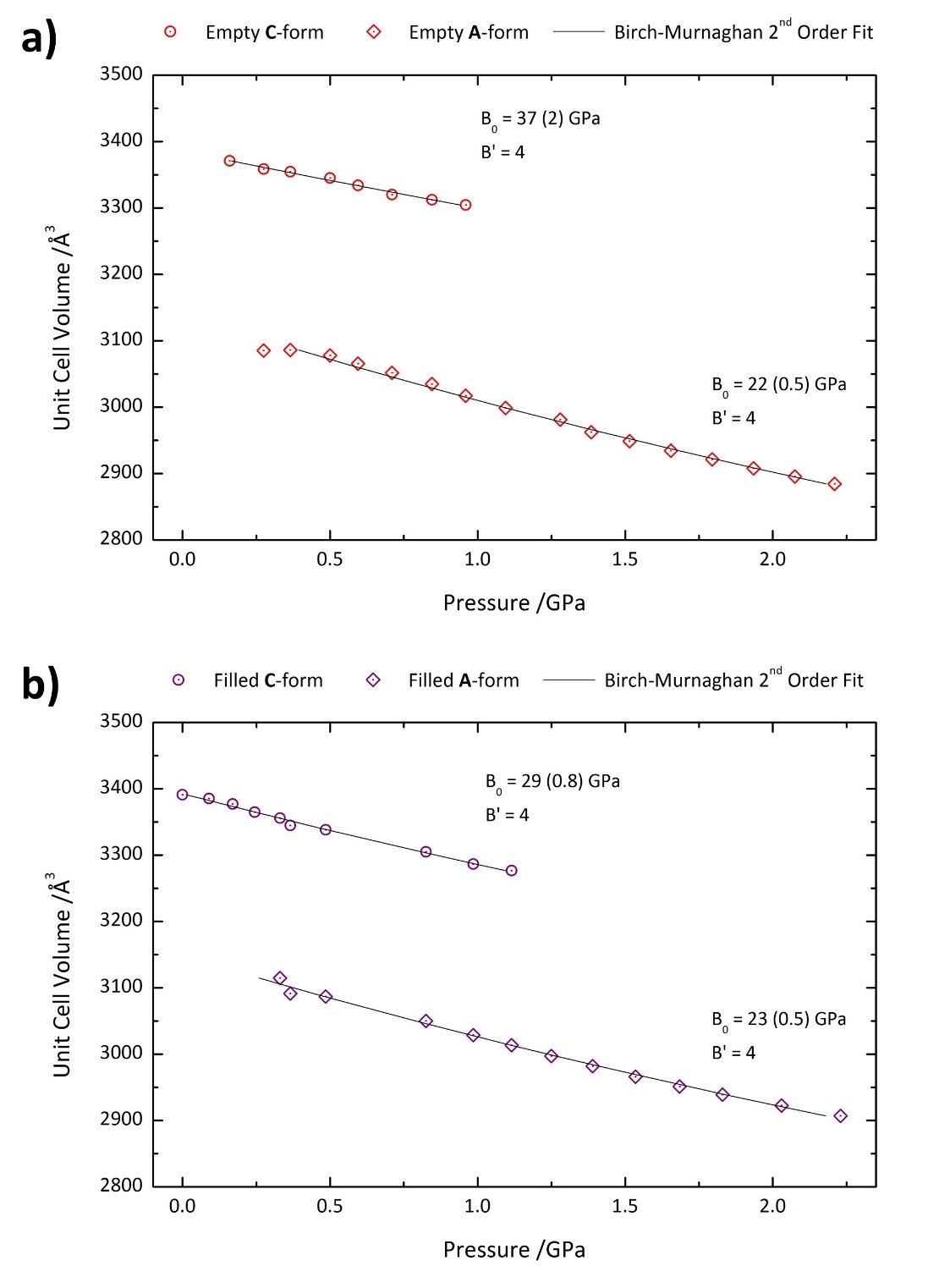


**Figure S20.** The variation in unit cell volume with pressure for a) the empty and b) the filled zeolite RHO samples, in the 0-2.2 GPa pressure range. Shown are the Birch-Murnaghan 2^nd^ order fits, bulk moduli (B_0_) and pressure derivatives (B’) determined using the webtool PASCal [47] for both the C and A-forms observed. The C-form is shown with circle data points, and the A-form with diamonds.


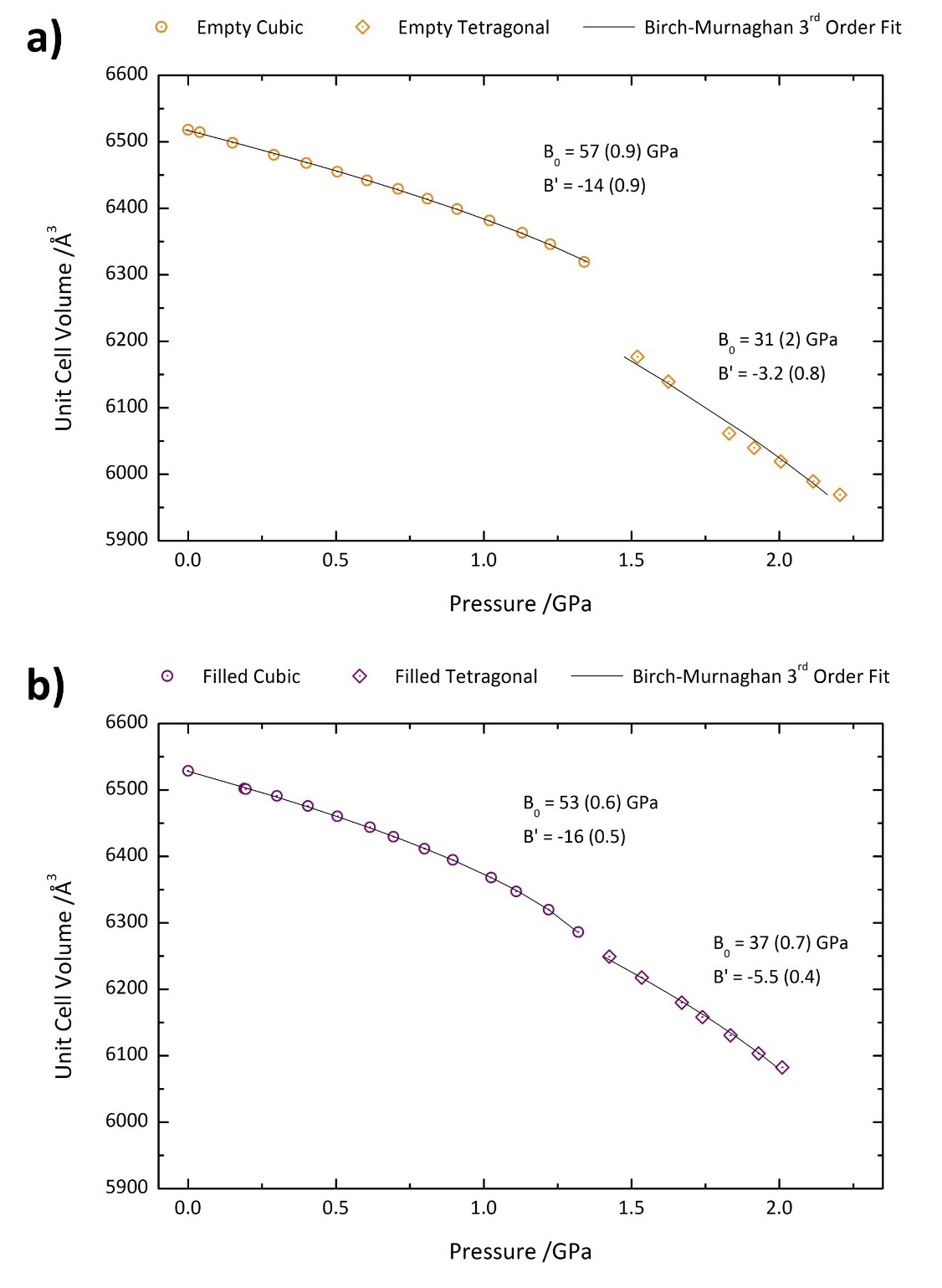


**Figure S21.** The variation in unit cell volume with pressure for a) the empty and b) the filled zeolite ZK-5 samples, in the 0-2.2 GPa pressure range. Shown are the Birch-Murnaghan 3rd order fits, bulk moduli (B_0_) and pressure derivatives (B’) determined using the webtool PASCal [47] for both the cubic and tetragonal phases observed. The cubic phase is shown with circle data points, and the tetragonal phase with diamonds.
